# A secretory phospholipase D hydrolyzes phosphatidylcholine to suppress rice heading time

**DOI:** 10.1371/journal.pgen.1009905

**Published:** 2021-12-08

**Authors:** Li Qu, Yu-Jia Chu, Wen-Hui Lin, Hong-Wei Xue

**Affiliations:** 1 Shanghai Collaborative Innovation Center of Agri-Seeds, Joint Center for Single Cell Biology, School of Agriculture and Biology, Shanghai Jiao Tong University, Shanghai, China; 2 National Key Laboratory of Plant Molecular Genetics, CAS Center for Excellence in Molecular Plant Sciences, Chinese Academy of Sciences, Shanghai, China; 3 School of Life Sciences and Biotechnology, The Joint International Research Laboratory of Metabolic and Developmental Sciences, Joint Center for Single Cell Biology, Shanghai Jiao Tong University, Shanghai, China; National University of Singapore and Temasek Life Sciences Laboratory, SINGAPORE

## Abstract

Phospholipase D (PLD) hydrolyzes membrane phospholipids and is crucial in various physiological processes and transduction of different signals. Secretory phospholipases play important roles in mammals, however, whose functions in plants remain largely unknown. We previously identified a rice secretory PLD (spPLD) that harbors a signal peptide and here we reported the secretion and function of spPLD in rice heading time regulation. Subcellular localization analysis confirmed the signal peptide is indispensable for spPLD secretion into the extracellular spaces, where spPLD hydrolyzes substrates. *spPLD* overexpression results in delayed heading time which is dependent on its secretory character, while suppression or deficiency of *spPLD* led to the early heading of rice under both short-day and long-day conditions, which is consistent with that *spPLD* overexpression/suppression indeed led to the reduced/increased Hd3a/RFT1 (*Arabidopsis* Flowing Locus T homolog) activities. Interestingly, rice Hd3a and RFT1 bind to phosphatidylcholines (PCs) and a further analysis by lipidomic approach using mass spectrometry revealed the altered phospholipids profiles in shoot apical meristem, particularly the PC species, under altered *spPLD* expressions. These results indicate the significance of secretory spPLD and help to elucidate the regulatory network of rice heading time.

## Author summary

Secretory phospholipases play essential roles in physiological processes of mammals, while functions of them in plants remain unknown. We identified a rice secretory PLD (spPLD) harboring a signal peptide which is indispensable for secretion of spPLD. Functional studies showed that altered *spPLD* expression resulted in the changed heading time of rice under both short-day and long-day conditions, which is dependent on the secretory character of spPLD. Rice Hd3a and RFT1, the homologs of *Arabidopsis* Flowing Locus T (FT), bind to phosphatidylcholine (PC) to promote heading. Analysis of phospholipids profiles in shoot apical meristem by using a mass spectrometry-based lipidomic approach demonstrated that spPLD regulates heading time by hydrolyzing the light period-predominant PC species, further revealing the crucial role of secretory proteins in regulating plant growth and development.

## Introduction

Phospholipase Ds (PLDs) form a major family of phospholipases and with its derived product phosphatidic acid (PA) involve in regulation of various physiological and cellular processes of plant growth and development, as well as plant response to environmental stimuli [1–4]. PLDs can be grouped into different classes based on gene structure, domain organization, sequence similarity and biochemical properties in *Arabidopsis* and rice [5,6]. Interestingly, protein domain structural analysis revealed the presence of a special class of PLDs known as spPLD [5], which was firstly identified in rice genome and harbors a signal peptide at N-terminus instead of a C2 domain or PX/PH domain. Homolog of spPLD was also identified in grapes and poplar by sequence analyses, however, the physiological functions of them are still unknown [7]. Although genetic studies have demonstrated the crucial roles of PLDs in multiple physiological processes including lipid degradation [8], vesicular tracking [9,10], plant innate immunity [11], hormone effects and stresses responses [12], the secretory character and physiological functions of the secretory PLD in plants remains unclear.

In mammalian cells, phospholipase D2 (PLD2) localizes at rims of Golgi complex and regulates the constitutive secretion in epithelial cells and glycoconjugate trafficking in mast cells [13], thus involves in plethora of cellular functions including cell signaling [14], apoptosis [15] and cancer [13]. Studies showed that overexpressed PLD2 is secreted by colon tumor cells and changes the microenvironment to increase stem cell fate of tumor cells by inducing senescence in neighboring fibroblasts [16], which demonstrates the importance of secretion in PLD2 function. Compared to the reported secretory PLD in mammalian cells, there are few annotation and functional studies of secretory PLD in plants yet.

Flowering marks the growth transition from vegetative stage into reproductive stage, and time of flowering determines whether plants are able to produce sufficient seeds to extend the life cycle [17], as well as contributes the distribution, yield and quality of crops. Florigen is a systemic signal to induce floral transition at shoot apical meristem (SAM) after being transported from leaves [18–20]. In *Arabidopsis*, Flowing Locus T (FT) protein is the florigen [18] and in rice, Heading date 3a (Hd3a) and Rice Flowering Locus T 1 (RFT1) are two confirmed florigens mainly response for heading regulation under short-day (SD) or long-day (LD) condition, respectively [21,22,23]. By identifying various mutants, systemic genetic studies have identified the main pathway and components controlling flowering time in *Arabidopsis* [24,25], however, whether phospholipid molecules regulate flowering time remained elusive until recent studies using *Arabidopsis* revealed that FT preferentially interacts with diurnally changing phosphatidylcholine (PC) species in SAM, leading to enhanced FT activity and hence promoted flowering [26]. A significantly higher resolution crystal structure of FT was determined and the putative binding sites for phosphatidylcholine (PC) were predicted with computational docking simulation [27]. In addition, studies showed that FT interacts with the negatively charged phosphatidylglycerol (PG) to regulate temperature-insensitive early flowering [28]. However, whether phospholipid molecules regulate heading date in rice as well remains unknown.

Here, we showed the secretory character of rice spPLD and uncovered its conserved function in regulating flowering time in both rice and *Arabidopsis*. Analysis of phospholipids profiles in rice SAM confirmed the significantly altered PC contents under changed *spPLD* expression, providing a mechanistic insight into how secretory spPLD regulates heading time of rice through modulating the light period predominant PC species and Hd3a/RFT1 activities.

## Results

### spPLD is a secretory PLD in plants

**Fig 1:**
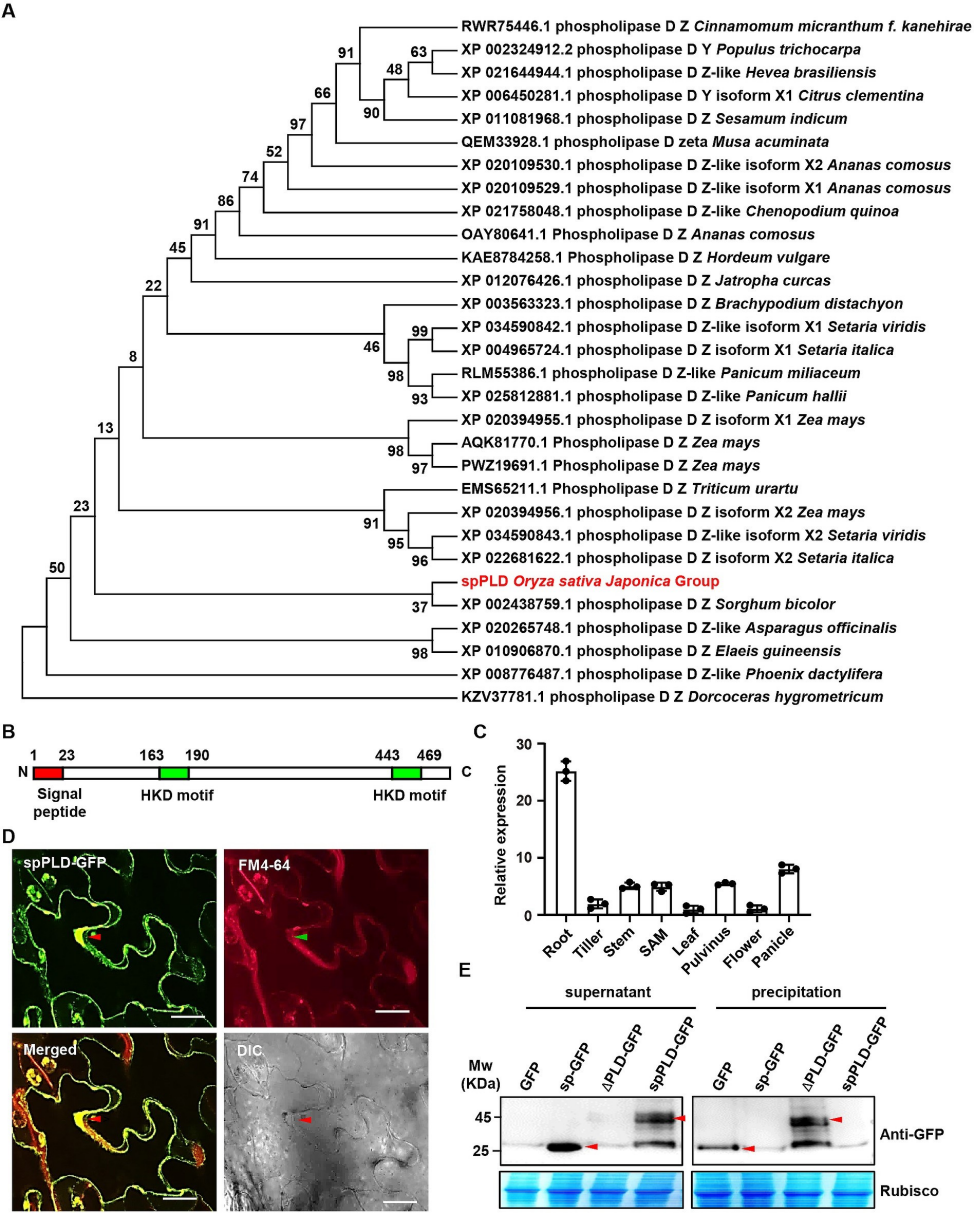
spPLD is a secretory PLD. A. Sequence alignment was performed with BLAST and the top 30 proteins with protein functional annotation and identity at more than 70% were analyzed. The bootstrap consensus tree inferred from 1000 replicates is generated to represent the evolutionary history of the taxa analyzed. The percentage of replicate trees in which the associated taxa clustered together in the bootstrap test (1000 replicates) is shown next to the branches. NCBI accession number, protein ID and species of analyzed protein are shown. B. Protein structural analysis showed the presence of two HKD motifs and one signal peptide (sp) at N-terminus of rice spPLD. C. qPCR analysis revealed the *spPLD* expression in various tissues including roots, tillers, stems, SAM, leaves, pulvini, flowers and panicles. Expression level was normalized to *ACTIN1* transcript and relative expressions were calculated by setting *spPLD* expression in leaves as 1.0. Experiments were repeated three times and data were shown as mean ± SD (n = 3). D. Secretion of spPLD was confirmed by observing *N*. *benthamiana* plants expressing spPLD-GFP fusion protein. FM4-64 was used to highlight the plasma membrane. Plasmolysis was conducted by 1 M mannitol treatment for 10 min. The secreted sections were highlighted by arrows. DIC, bright field. Scale bar = 50 μm. E. Western Blotting analysis confirms the secretion of spPLD. Various fusion proteins were transiently expressed in rice protoplasts. After incubation for 48 h, proteins of supernatant (incubation medium) and precipitation (protoplast homogenate) were extracted and analyzed by Western Blotting using anti-GFP antibody. Arrows highlighted the GFP (sp-GFP) and spPLD-GFP (ΔPLD-GFP) proteins.

Given the importance of secretory PLDs in animals, it is supposed that spPLD may also play an important role in regulating rice growth and development. Phylogenetic analysis of the top 30 proteins with >70% identities by functional annotation showed that spPLD is in an evolutionarily separated branch (Fig 1A). Structural analysis revealed the presence of signal peptide (sp) at N-terminus, and two highly conserved HKD motifs [H(X)K(X)_4_D] at middle and C-terminus, of spPLD (Figs 1B and S1). Further phylogenetic analysis showed the uniqueness of signal peptide (S2 Fig), while HKD motifs are highly conserved in various phospholipases (S3 and S4 Figs). Quantitative RT-PCR (qPCR) analysis showed that *spPLD* is transcribed in various tissues, with a relative higher expression in roots and panicles (Fig 1C). Further analysis using RiceXPro (https://ricexpro.dna.affrc.go.jp/) showed that *spPLD* is highly transcribed in leaf during vegetative period especially before 34 days after transplanting (DAT, S5A Fig), and interestingly, presents diurnal oscillation (lowest in the morning and highest in the evening, S5B Fig).

To investigate the role of signal peptide, we observed tobacco leaves transiently expressing spPLD-GFP fusion protein and found that spPLD-GFP is localized between plasma membrane and cell wall (FM4-64 staining was applied to mark the plasma membrane), suggesting the secretion of spPLD (Fig 1D). Further detailed observation of various GFP fusion proteins showed the localization between plasma membrane and cell wall of spPLD-GFP and signal peptide-GFP (sp-GFP), while removal of signal peptide (ΔPLD-GFP) resulted in the fluorescence limited to cytoplasm (S5C Fig). Additionally, we transiently expressed the fusion proteins in rice protoplast and analyzed the protein accumulation in incubation medium and protoplast homogenate. Results showed that most spPLD-GFP existed in the supernatant, as well as GFP under signal peptide of spPLD, while ΔPLD-GFP was detected in the precipitation only (Fig 1E), confirming that spPLD protein is secreted and soluble. All these results indicated the secretory character of spPLD and a crucial role of signal peptide in guiding the secretion of spPLD protein.

### spPLD delays rice heading time

**Fig 2:**
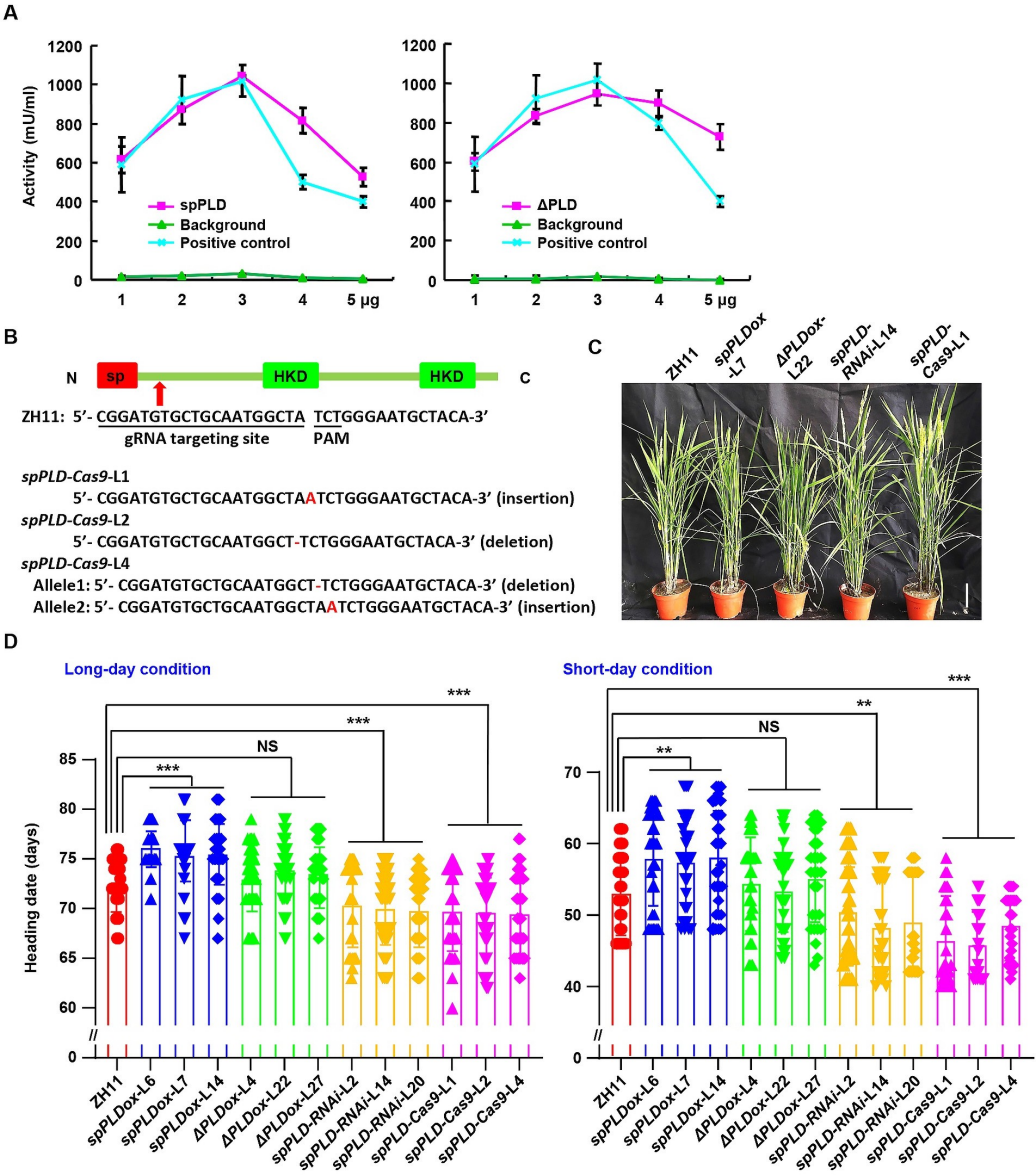
spPLD delays rice heading time. A. Enzymatic assay showed that both spPLD and ΔPLD (spPLD deleting the signal peptide) present PLD activity. Purified spPLD or ΔPLD (1–5 μg) proteins were used for examination and choline was used as substrate. There is no choline in background (as negative control) and positive control is supplied in assay kit. Experiments were repeated three times and data were shown as mean ± SD (n = 3). B. Sequencing confirmed three mutation lines of *spPLD* (insertion or deletion of bases), by CRISPR/Cas9. The gRNA targeting site and PAM sequence, and the position of gRNA at *spPLD* gene, are indicated. C. Phenotypic observation of rice plants with altered *spPLD* expression under natural long-day condition at heading stage. Representative images were shown. Scale bar = 10 cm. D. Rice plants with altered *spPLD* expression were grown under natural long-day (left) or short-day (right) conditions and heading date were calculated and statistically analyzed using Tukey’s test (**, *p* < 0.01; ***, *p* < 0.001). NS, no significance. Days to flowering were scored when first panicle was bolted, and data were shown as mean ± SD (n = 50).

*In vitro* enzymatic analysis demonstrated that both spPLD and ΔPLD presents hydrolyzing activity on choline (Fig 2A), indicating spPLD a functional PLD and whose activity is signal peptide independent. In eukaryotic cells, phospholipids are asymmetrically distributed on the lipid bilayer of plasma membrane and organelle membranes [29] and coincidentally, phosphatidylcholine (PC) and sphingomyelin (SM) are exposed on the cell surface [30,31], which shed light on the specific location and physiological effects of spPLD by hydrolyzing PC and indicate the significance for the secretory character of spPLD.

To investigate the physiological functions of spPLD, transgenic rice plants overexpressing *spPLD* (*spPLDox*) or *spPLD* removing the signal peptide (*ΔPLDox*) were generated (confirmed by qPCR, S6 Fig). Reduced expression of *spPLD* was achieved by either RNA interference-mediated silencing (*spPLD-RNAi*, confirmed by qPCR, S6 Fig) or CRISPR/Cas9 approach (*spPLD-Cas9*, confirmed by sequencing, Fig 2B). Phenotypic observation of homozygous lines showed that altered expression of *spPLD* does not lead to the obvious growth change during vegetative stage, while interestingly, compared with wild-type plants, significantly delayed heading date was observed in *spPLDox* plants, whereas *spPLD-RNAi* and *spPLD-Cas9* plants presented earlier heading, under long-day (LD) condition in paddy field (Figs 2C, 2D and S7A). Similar phenotype was also observed under short-day (SD) condition (Figs 2D and S7B), indicating that spPLD regulates heading time was not dependent on photoperiod. Interestingly, *ΔPLDox* plants don’t show delayed heading (Figs 2C, 2D and S7), which may due to the deficiency of signal peptide and thus the secretory character of spPLD, and suggests the dependence of spPLD function on the secretion. All these results indicate that spPLD is involved in the regulation of floral transition and functions after being secreted into apoplast.

### spPLD modulates contents of light period predominant PCs in SAM

**Fig 3:**
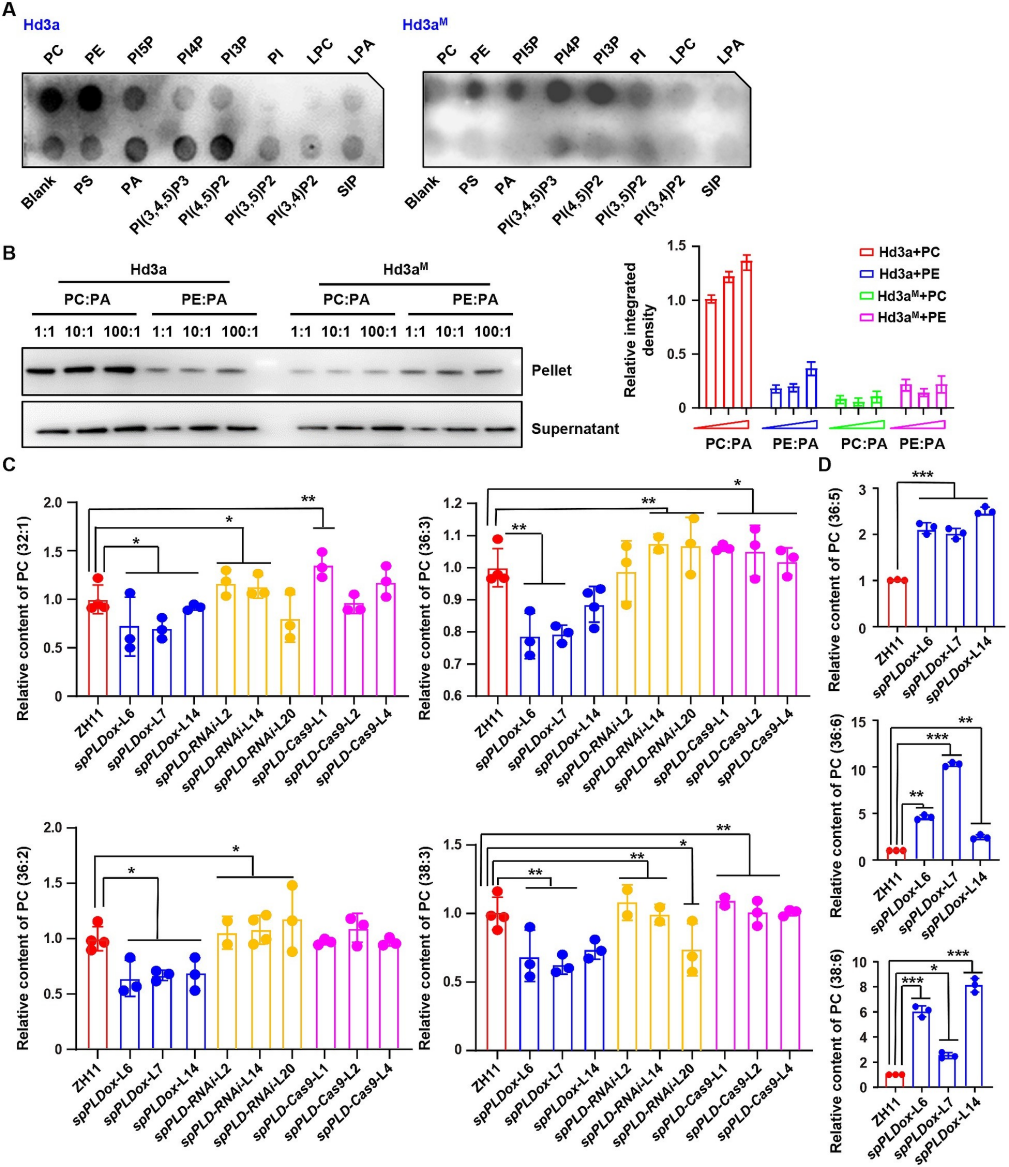
Altered PC species under *spPLD* overexpression or deficiency. A. Fat-western immunoblot analysis revealed the binding of rice Hd3a (left) and Hd3a with mutated PC binding sites (Hd3a^M^, right) to phospholipids. The phospholipid type of each dot is indicated. PC, phosphatidylcholine; PE, phosphatidylethanolamine; PS, phosphatidylserine; PA, phosphatidic acid; LPA, lysophosphatidic acid; LPC, lysophosphatidylcholine; S1P, sphingosine 1-phosphate; PI, phosphatidylinositol; PI3P, PI 3-monophosphate; PI4P; PI5P; PI(3,4)P_2_, PI 3,4-bisphosphate; PI(3,5)P_2_; PI(4,5)P_2_; PI(3,4,5)P_3_, phosphatidylinositol 3,4,5-trisphosphate. B. Liposome binding assay (left) and quantitative analysis (right) confirmed the preferential binding of Hd3a to PC. His-tag fused Hd3a and Hd3a with mutated PC binding sites (Hd3a^M^) were purified and incubated with liposomes containing different PC:PA or PE:PA ratios. After collecting the liposomes, the portion of proteins bound to liposomes was detected by western blotting using anti-His antibody. Nonbinding protein was detected in the supernatant (bottom). Band density is measured by Image J and relative density was calculated by setting the intensity under PC:PA ratio 1:1 as 1.0. Data were presented as means ± SD (n = 3, right). C-D. Relative content of predominant PCs with different saturation status, PC (32:1), PC (36:2), PC (36:3) and PC (38:3) (C); PC (36:5), PC (36:6) and PC (38:6) (D), in ZH11 and various lines with altered *spPLD* expressions. Ten rice shoot apical meristem before bolting were collected and used for lipids extraction. Phospholipids were profiled by a lipidomic approach using mass spectrometry. Experiments were biologically repeated three times and data were shown as mean ± SD (n = 3). Statistical analysis was performed by Tukey’s test (*, *p* <0.05; **, *p* < 0.01 ***, *p* < 0.001, compared to ZH11).

As an important developmental stage and agricultural trait, heading date is determined by both endogenous signals and environmental cues and recent studies showed that lipid-mediated signaling has emerged as one of the major regulatory pathways [32]. In *Arabidopsis*, the key factor regulating plant flowering, Flowering Locus T (FT), preferentially interacts with phosphatidylcholine (PC) species containing less unsaturated fatty acids (36:4, 36:3, 36:2, 36:1 and 34:1) that are predominant in light period to promote flowering [25]. Based on the similar phenotype under both SD and LD conditions, we examined the binding of rice Heading date 3a (Hd3a) and Rice Flowering Locus T 1 (RFT1), with phospholipids through Fat-western immunoblot analysis. Results revealed that both Hd3a and RFT1, bind to phospholipids as well (Figs 3A and S8A). Considering the revealed binding sites of FT for PC (R13, D17, R83 and R119) [27] and high homology of FT, Hd3a and RFT1, the Hd3a or RFT1 with mutated PC binding sites (Hd3a^M^ and RFT1^M^, the R or D of binding site were mutated to A) were generated. Examination of the phospholipids binding showed the significantly decreased PC binding ability of Hd3a^M^ or RFT1^M^ (Figs 3A and S8A).

Further liposome protein association assay showed that along with the increased ratio of PC:PA in liposomes, stronger binding of Hd3a/RFT1 to PC was observed, while the binding of Hd3a^M^/RFT1^M^ to PC weakened significantly (Figs 3B and S8B), confirming the binding ability of Hd3a/RFT1 to PC and indicating both Hd3a and RFT1 are also PC-binding proteins. Interestingly, in addition to PC, Hd3a and RFT1 bind to phosphatidylethanolamine (PE), however, liposome protein association assay showed their binding to PE was much weaker than PC and the binding with PE was not affected by the mutation of PC binding sites (Figs 3B and S8B), indicating the binding specificity and that Hd3a and RFT1 preferentially bind to PC than PE. In addition, Hd3a binds to various phospholipids including PI5P, PI(4,5)P_2_, PI(3,4,5)P_3_ and slightly binds to phosphatidic acid (PA), suggesting a similar and distinct regulatory mechanism of Hd3a/RFT1 by phospholipids.

Considering spPLD functions at apoplast to affect the heading time and PCs are distributed on the outer plasma membrane, whether altered spPLD expression resulted in the changed content of PC was examined by mass spectrometric analysis. Phospholipids profiles of shoot apical stem (SAM, ~ 7 days before bolting) of ZH11 and various transgenic lines under natural SD condition were investigated with a lipidomics approach [33]. Results showed that there was no evident change of content of Hd3a/RFT1-binding phospholipids, PC and PE, in transgenic lines with altered *spPLD* expressions (S9A and S9C Fig), while the content of PA was reduced in all examined lines (S9B Fig), suggesting a possible feedback regulation to reduce the PA amount since PA is the product of lipid hydrolysis which is a reversible process [1]. Although other phospholipids including PG, PI, PS, LPA and LPC displayed irregular changes (S9 Fig), there is no binding of them with Hd3a/RFT1 and considering the subcellular location of these phospholipids and secretory character of spPLD, it is suggested that these phospholipids might be not directly involved in the heading time regulation through Hd3a/RFT1.

Although there was no significant difference of total PC levels in rice lines with altered expressions of *spPLD*, considering the roles of distinct PC species in regulating FT activity and flowering, content of different PC species, particularly with different saturation status, were detailed analyzed. Results indeed showed the evident alteration of specific PC species. Contents of less unsaturated PC species (32:1, 36:2, 36:3 and 38:3) that are predominant in light period are decreased in *spPLDox*, while increased in *spPLD-RNAi* or *spPLD-Cas9* lines (Figs 3C and S10A–S10D); the contents of high unsaturated PC species (36:5, 36:6 and 38:6) that are predominant in night period are not altered in *spPLD-RNAi* or *spPLD-Cas9* lines, whereas significantly increased in *spPLDox* lines (Figs 3D and S10E–S10G), which may have a close correlation with the diurnal rhythmic expression of *spPLD*.

**Fig 4:**
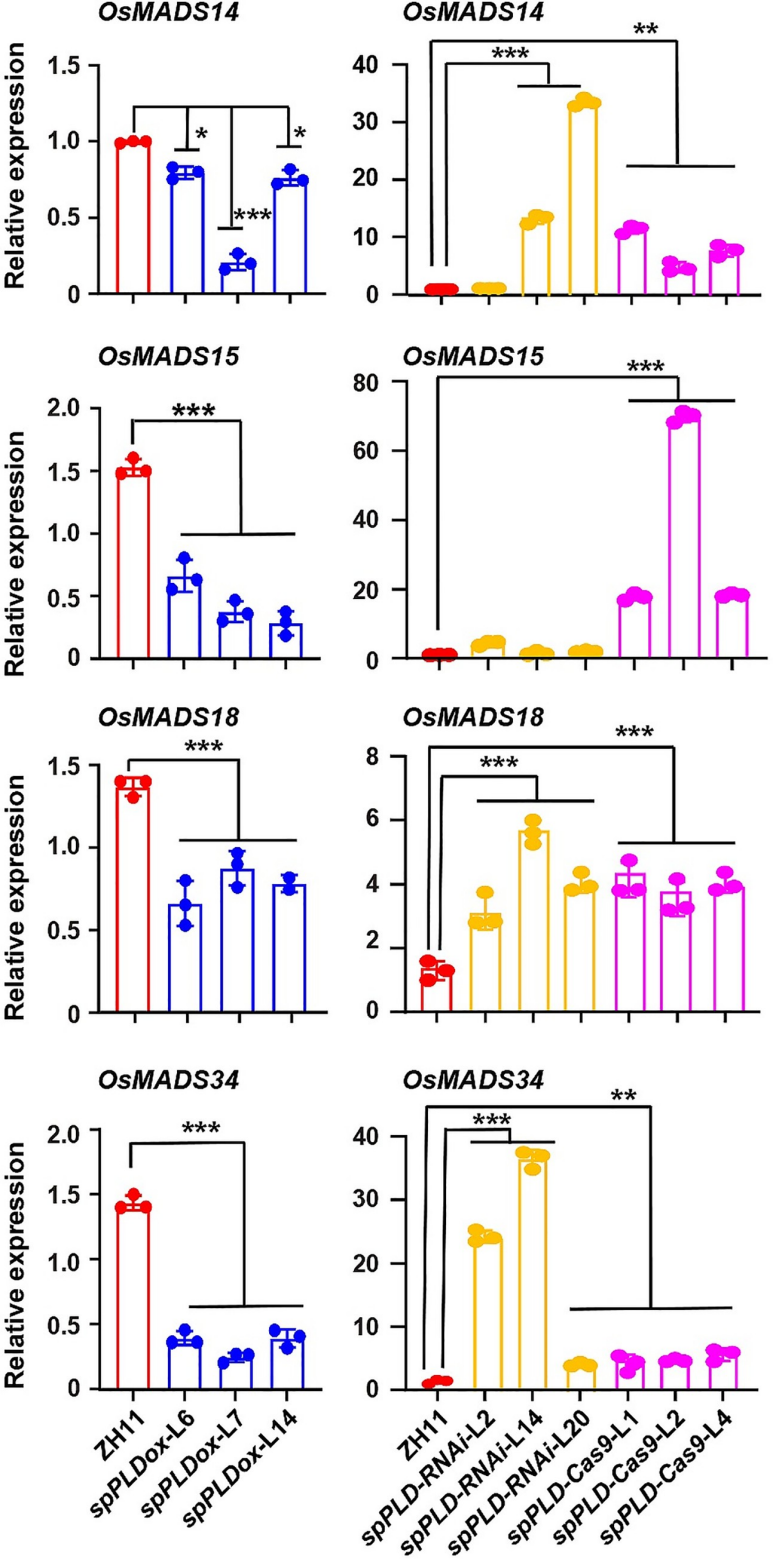
Expression of *OsMADS14*, *15*, *18* and *34* were decreased in *spPLDox* lines while increased in *spPLD-RNAi/Cas9* lines. Total RNAs were extracted from SAM (~ 1 cm in length) of five-week-old rice plants (~ 7 days before bolting) and expressions of *OsMADSs* genes were examined by qPCR analysis. Rice plants were grown under LD condition (14-h light / 10-h dark cycle, 28°C) with 70% humidity. Expression levels of examined genes were normalized to *ACTIN1* transcript. Experiments were repeated for three times and data were shown as mean ± SD (n = 3). Statistical analysis was performed by Tukey’s test (*, p <0.05; **, p < 0.01; ***, p < 0.001, compared to ZH11).

*Arabidopsis* FT preferentially interacts with less unsaturated PC species (particularly 36:2 and 36:3) to promote flowering, while high unsaturated PC species delay flowering [26]. Hd3a and RFT1 bind PCs and further examination of the expressions of Hd3a/RFT1 downstream genes (*OsMADS14/15/18/34*) by qPCR showed that the transcription of them were significantly decreased in *spPLDox* lines while increased in *spPLD-RNAi* or *spPLD-Cas9* lines (Fig 4), indicating that decreased Hd3a/RFT1’ activity in *spPLDox* lines maybe the direct and main reason for delayed heading. In addition, Hd3a functions as a mobile signal and promotes branching through lateral bud outgrowth [34]. Analysis of the tiller numbers consistently showed the significantly increased tillers of *spPLD-RNAi* or *spPLD-Cas9* rice lines (S11A Fig), which confirmed the enhanced Hd3a activity under deficiency/suppression of *spPLD*.

**Fig 5:**
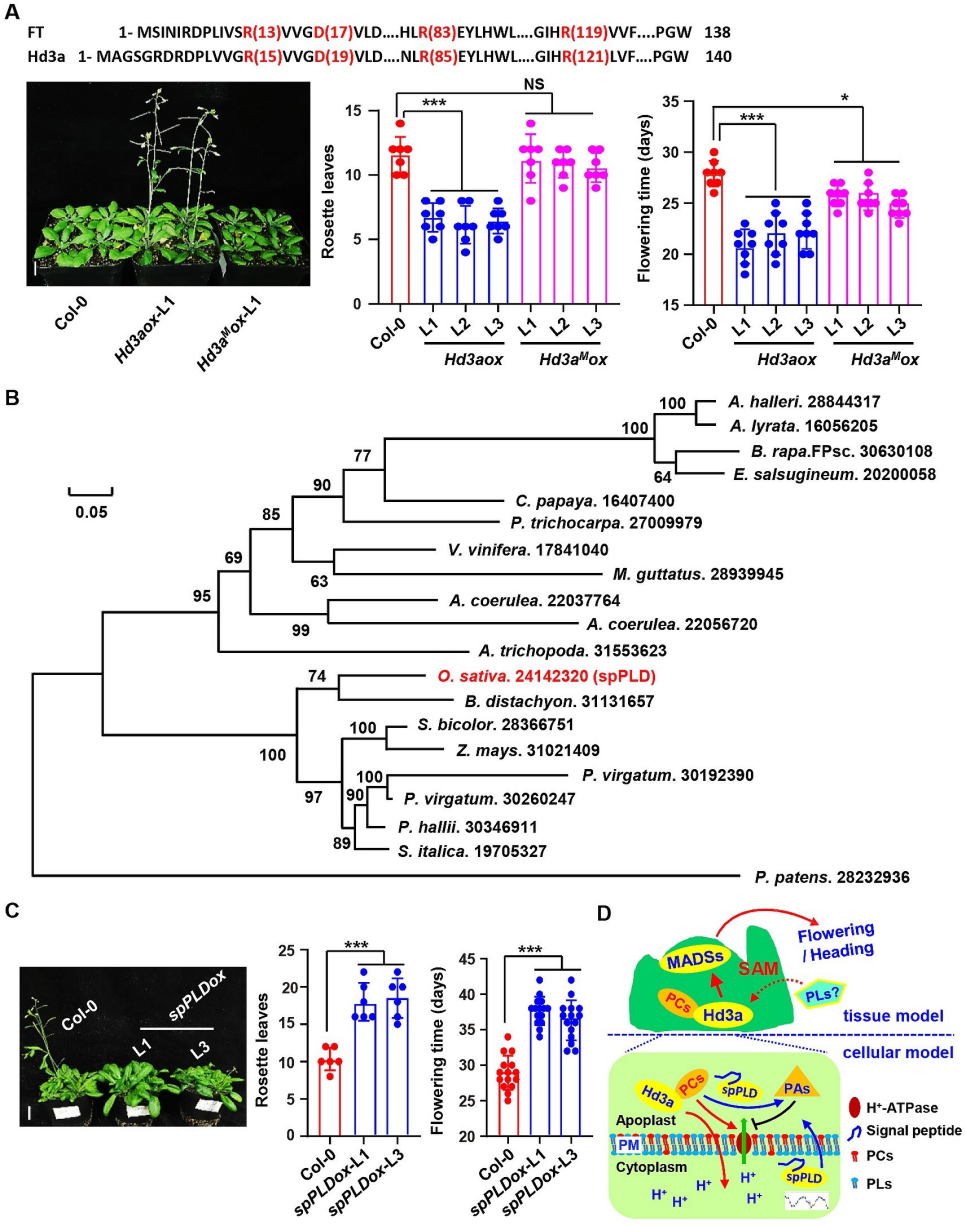
Conserved function of spPLD. A. Hd3a with mutated PC binding sites (Hd3a^M^) did not promote flowering time of *Arabidopsis* as Hd3a. Transgenic *Arabidopsis* overexpressing Hd3a showed early flowering under long-day condition while those overexpressing Hd3a^M^ did not (left). Number of rosette leaves and flowering time of plants were calculated (right) and statistically analyzed by Tukey’s test (lower, *, *p* < 0.05; ***, *p* < 0.001, compared to Col-0). NS, no significance. Representative images were shown (left, bar = 1 cm) and data were shown as mean ± SD (n = 15). Protein sequence of *Arabidopsis* FT and rice Hd3a were shown, and amino acids for PC binding of *Arabidopsis* FT, which is conserved in rice Hd3a and used for mutation analysis, were highlighted by red color (upper). B. Phylogenetic analysis of spPLDs in different plant species. Sequence alignment and the rectangular cladogram were generated with ClustalX 2.0 or TreeView respectively. Amino acid pairwise identity (percentage) between different protein sequences was shown. Scale bar represents 0.05 amino acid substitutions per site. C. spPLD delays flowering time of *Arabidopsis*. Transgenic *Arabidopsis* overexpressing *spPLD* showed delayed flowering under long-day condition. Number of rosette leaves and flowering time of plants were calculated and statistically analyzed using Tukey’s test (right, ***, *p* < 0.001, compared to Col-0). Representative images were shown (left, bar = 1 cm) and data were shown as mean ± SD (n = 15). D. A hypothetical model (tissue model and cellular model respectively) illustrating how secretory spPLD functions in suppressing rice heading time. spPLD showing a diurnally rhythmic and JA-upregulated expression, is secreted and hydrolyzes phosphatidylcholine (PC) at apoplast to reduce the levels of light period predominant PC species (less unsaturated PCs) in shoot apical meristem (SAM), which may lead to the reduced activity of primary pump (H^+^-ATPase) at plasma membrane, resulting in the less driving force for Hd3a/RFT1 entering cell at SAM, hence the decreased Hd3a/RFT1 activity and delayed heading. Other phospholipids may involve in heading time regulation through binding with and regulating the activity of Hd3a/RFT1.

To further explain whether the Hd3a-phospholipid interaction is necessary to promote the heading, transgenic *Arabidopsis* overexpressing Hd3a (*Hd3aox*) and Hd3a^M^ (*Hd3a*^M^*ox*) were generated (confirmed by qPCR, S12 Fig). Phenotypic analysis showed that compared to that *Arabidopsis* seedlings overexpressing Hd3a presented early flowering, seedlings overexpressing Hd3a^M^ did not show promoted flowering time as those overexpressing Hd3a under LD condition (Fig 5A), indicating binding with PC is important for Hd3a function in promoting flowering time.

### Conserved function of spPLD in plants

Sessile plants face various unfavorable environments throughout life cycle and respond advantageously to reduce damages such as delayed flowering by increasing biomass accumulation for survival. To further explore the conservation of spPLD in plants, phylogenetic analysis of spPLD with focus on several monocotyledons and dicotyledons indicating that spPLD homolog not only exists in monocotyledons but also dicotyledons including *Arabis halleri* and *Arabidopsis lyrata* (Fig 5B). *Arabidopsis* transgenic lines overexpressing *spPLD* were then generated (S12 Fig) and observation indeed revealed the delayed flowering of these lines (Fig 5C), suggesting a conserved function of spPLD in both monocotyledons and dicotyledons. Considering the absence of spPLD homolog in *Arabidopsis*, there may exist a substitute protein during evolution to regulate floral transition after being secreted into apoplast to hydrolyze PC and hence to suppress FT activity.

## Discussion

Flowering/heading time is a key agronomic trait that determines cropping season and regional adaptability [35], hence an important agricultural trait for crop (such as rice) breeding. Lipid-mediated signaling has been shown to play crucial roles in regulating flowering time [26,28]. By combining functional genomics and systemic phospholipids profile analysis, our studies suggested that a secretory spPLD suppresses rice heading date through decreasing the content of light period predominant PC species, which leads to the reduced activity of primary pump (H^+^-ATPase) at plasma membrane and less driving force for Hd3a/RFT1 entering cell, hence the decreased Hd3a/RFT1 activity and delayed heading (Fig 5D), revealing the importance of a secretory spPLD in the lipid-mediated regulation of heading time in rice.

Compared to plants overexpressing *ΔPLD*, significantly delayed heading was observed in those overexpressing *spPLD*, indicating the necessity of signal peptide and demonstrating the importance of secretion in spPLD function. These provide evidence for the important roles of secretory proteins in regulating plant growth and development and confirm the crucial role of secretion in effect of secretory proteins. The important role of spPLD in regulating heading time was further confirmed by the early heading of *spPLD-RNAi* or *spPLD-Cas9* lines and the conserved function in *Arabidopsis*.

In addition to binding with PC, a lipidomic approach analysis of phospholipids profiles in rice SAM showed the significant difference of distinct PC species under altered *spPLD* expression, further suggesting that spPLD may affect PCs level especially the light dominant species through secretion thus to regulate heading time, which is consistent with the diurnal oscillation of *spPLD* transcription. Hd3a and RFT1 are mobile signals transporting to cells in shoot apex [20], the binding with less unsaturated PC species at apoplast may stimulate the entering of Hd3a and RFT1 to the cell. Although Hd3a and RFT1 also bind to PE with weaker binding ability, the different location of PC and PE at cell membrane (the PCs exposed on the outer plasma membrane [30,31]) and spPLD functions dependent on secretion indicated that PC is mainly involved in heading time by facilitating the entering of Hd3a/RFT1 to the cell, hence promoting the Hd3a/RFT1 signaling.

The plasma membrane of plant cells contains the H^+^-ATPase, which functions as the most important primary pump to produce a proton electrochemical gradient across plasma membrane providing the driving force for active transport into cells [36]. Previous studies showed that in rice culture cells H^+^-ATPase activity is stimulated by PC and inhibited by PA [37,38]. It is thus hypothesized that in rice plants, overexpressed *spPLD* hydrolyzes PC into PA at the apoplast, resulting in the reduced levels of PC species and increased PAs in SAM. Altered phospholipids leads to the decreased H^+^-ATPase activity and less driving force for Hd3a/RFT1 entering cell, resulting in the reduced Hd3a/RFT1 activity and late heading (Fig 5D), which is consistent that plants overexpressing *ΔPLD* did not show delayed heading.

Previous microarray-based expression profile analysis revealed a specific expression of *spPLD* compared with other rice *PLDs*, i.e. being downregulated under abiotic stress conditions [39], while analysis with Transcriptome Encyclopedia Of Rice (https://tenor.dna.affrc.go.jp/) showed that *spPLD* expression is upregulated by Jasmonic acid (JA) in shoot. Detailed analysis by qPCR showed that JA indeed stimulates *spPLD* expression (S13 Fig), which suggests a possible role of spPLD in plant stress responses and is consistent with the study that JA signaling is involved in floral transition regulation in *Arabidopsis* through JA receptor COI1, JA-ZIM domain (JAZ) proteins and JA-activated transcription factors [40]. JA acts as a crucial defense signal and mainly regulates plant resistance against pathogens and insects, and defense responses are often accompanied by significant growth inhibition including flowering [41,42].

As a kind of glycerolipid, PCs function in multiple physiological processes depending on its acyl composition which continuously changes according to varying environments such as light, temperature and atmospheric constituents [43,44]. Interestingly, PC molecular species present diurnal changes by a lipidomic profiling approach and FT preferentially interacts with light period predominant PC species at end of day to promote flowering in *Arabidopsis* [26]. Rice *Hd3a* is the homologous gene of *Arabidopsis FT* and promotes flowering under SD condition and *RFT1* is the closest homologue of *Hd3a* to promote heading under LD condition [19,23,45]. Crystallographic analysis revealed the PC binding sites of FT and showed that Hd3a may bind PCs and with similar molecules that FT binds [27,46], which is confirmed by Fat-western blot analysis and liposome protein association assay, particularly the mutation of PC binding sites reduced the PC binding ability of Hd3a and RFT1. Both Hd3a and RFT1 could bind PC, which is consistent with the changed heading date under both SD and LD conditions of transgenic lines with altered *spPLD* expression. It is speculated that, like *Arabidopsis* FT, rice Hd3a/RFT1 preferentially binds increased light period predominant PC species (36:2 and 36:3) in *spPLD-RNAi* and *spPLD-Cas9* lines to promote heading while conversely in *spPLD* overexpression lines.

PI-bisphosphates play a critical role in PLD activation in mammals, yeast and plants [6,47] and two putative PIP2-binding motifs flanking the second HKD domain of PLD are predicted [48]. In addition to PC, it is interesting that Hd3a and RFT1 bind to other phospholipids [PE, PI5P, PI(4,5)P_2_, PA] beside PCs, which is different from that of *Arabidopsis* FT and suggests the additional regulatory mechanism of Hd3a activities, i.e. by other phospholipids (and relevant enzymes) under distinct developmental or environmental signals. Indeed, endosomal trafficking is involved in FT movement and transport [49] and various phospholipids including PA and PI(4,5)P_2_ regulate the endosomal trafficking of target proteins through binding or regulating vesicle trafficking [4,50]. Indeed, a recent study revealed that negatively charged phospholipid PG could bind FT and affect the FT movement to regulate the temperature-insensitive flowering [28], which suggests that FT-phospholipid binding specificity may differ depending on the distinct localization and function [51]. How various phospholipids regulate Hd3a/RFT1 activity and hence flowering under different developmental or environmental factors need further investigation. In addition, *spPLD* is highly expressed in roots and overexpression of which results in the suppressed primary root length (S12B Fig), whether spPLD involves in root-related stress resistance, nutrition uptake by altering phospholipid content, for example PA, through secretion is worthy of further investigations.

## Materials and methods

### Plant materials and growth conditions

Rice Zhonghua 11 (ZH11, *Oryza sativa*, *japonica* cultivar) was used for transformation. Rice plants were grown in a paddy field in Shanghai under natural long-day (LD) condition or in Lingshui under natural short-day (SD) condition. For growth of transgenic plants, rice seeds were germinated in sterilized water and grown in a phytotron under a 12-h light (28°C)/12-h dark (22°C) cycle.

*Arabidopsis thaliana* (Columbia ecotype, Col-0) plants were used for transformation. Sterilized seeds of Col-0 and transgenic plants were stratified at 4°C for 2 days, then germinated on half-strength MS (1/2 MS) medium (Duchefa) under 22±1°C with a 16-h light/8-h dark photoperiod. Seven-day-old seedlings were transferred to soil and grown under identical conditions.

### Vector construction and transformation

To generate rice plants overexpressing spPLD or ΔPLD (spPLD removing signal peptide), coding regions of *spPLD* or *ΔPLD* were amplified by PCR (primers pUN1301-spPLD-LP/RP, pUN1301-ΔPLD-LP/RP). Resultant amplified fragment was digested with *Kpn*I and then subcloned into pUN1301.

To suppress *spPLD* expression, 80 bp DNA fragment of *spPLD* coding sequence was amplified by PCR (primers *spPLD-RNAi*-LP/RP) and subcloned into pMD-18T vector with both sense and antisense orientations. Resultant vector was then digested with *Sac*I / *Pst*I and obtained fragment was inserted into pCambia2301 to generate *spPLD-RNAi* construct.

To obtain *spPLD-Cas9* construct, a sgRNA (ATGTGCTGCAATGGCTATCT) driven by an OsU3 promoter and targeted to *spPLD* was inserted into the *Aar*I site of p2300-rCas9-U3-gRNA vector (primers *spPLD-Cas9*-LP/RP, the vector contains a rice-codon optimized Cas9 driven by a 2X CaMV35S promoter).

To generate *Arabidopsis* overexpressing *spPLD* or *ΔPLD*, *Hd3a* or *Hd3a*^*M*^, coding regions of *spPLD* or *ΔPLD* were amplified by PCR (primers AT-*spPLD*-PHB-LP/RP or AT-*ΔPLD*-PHB-LP/RP and AT- *Hd3a* -PHB-LP/RP or AT- *Hd3a*^*M*^-PHB-LP/RP). Resultant amplified fragment was digested with *Xho*I / *Xba*I and subcloned into PHB vector. All primers used are listed in S1 Table.

Generated constructs were confirmed by sequencing and transformed into *Agrobacterium tumefaciens* strain EHA105 for rice transformation using immature embryos as materials, or strain GV3101 for *Arabidopsis* transformation by floral dip method. Transgenic plants were selected by antibiotic resistance and confirmed by qPCR.

### RNA extraction and quantitative RT-PCR (qPCR) analysis

Total RNAs were extracted using Trizol reagent (Invitrogen) and reversely transcribed to first-strand cDNA. qPCR analysis was performed with Real-Time PCR Master Mix (Toyobo) and data were collected using the Bio-Rad Real Time detection system in accordance with the manufacturer’s instruction manual. Expressions of *spPLD* (primers RT-*spPLD*-LP/RP), *ΔPLD* (primers RT-*ΔPLD*-LP/RP), *Hd3a/Hd3a*^*M*^ (primers RT-*Hd3a*-LP/RP) and *OsMADSs* (primers RT-*OsMADS14/15/18/34*-LP/RP) were analyzed and normalized to that of rice *ACTIN1* or *Arabidopsis ACTIN2*. Primers are listed in S1 Table.

For methyl jasmonate (MeJA) treatment, rice seeds were germinated on a plastic mesh that were floated in a culture vessel on aseptic and deionized water at 28°C in a growth chamber for 10 days. Seedlings roots were soaked in JA (50 or 100 μM) for 1, 3, 6, 12 or 24 h. After treatment, the samples were immediately frozen in liquid nitrogen and used for RNA extraction and further analysis by qPCR.

### Database search and phylogenetic analysis

The spPLD homologues, signal peptide (sp) and HKD catalyze domains of spPLD in other species were searched by BLASTP program in NCBI databases (http://www.ncbi.nlm.nih.gov/). Phylogenetic analyses were performed through the alignments and the dendrograms were generated by the Maximum Likelihood method based on the JTT matrix-based model [52]. Evolutionary analyses were conducted in MEGA7 [53].

To verify the presence of a signal peptide in spPLD, analysis was conducted using SignalP databases (http://www.cbs.dtu.dk/services/SignalP/).

### Subcellular localization analysis

For analysis using onion epidermal cells, full-length cDNA of *spPLD* without stop codon, coding region of signal peptide (sp) or *spPLD* without signal peptide (ΔPLD) were amplified by PCR (primers *spPLD*-GFP-LP/RP, *sp*-GFP-LP/RP, *ΔPLD*-GFP-LP/RP) and subcloned into pA7-GFP. Resultant constructs were confirmed by sequencing and introduced into onion epidermal cells using a helium biolistic particle delivery system (Bio-Rad) according to previous description [54] (10 μg of plasmid for each bombardment). After incubation at 22°C in dark for 24 or 48 h, subcellular distribution of GFP fluorescence was observed by confocal laser scanning microscopy (Leica) with an argon laser excitation wavelength of 488 nm. A pA7 construct containing GFP was included as a control and plasmolysis was conducted by applying 1 M mannitol for 10 min. The bombardment experiments for each construct were biologically repeated at least three times, and each time at least 20 fluorescent onion epidermal cells were observed.

For analysis using tobacco leaves, full-length cDNA of coding region of *spPLD* without stop codon were amplified by PCR (primers UN1302-*spPLD*-LP/RP) and subcloned into PUN1302. Resultant constructs were confirmed by sequencing and transformed into *N*. *benthamiana* plants by agroinfiltration. After transformation for 3 days, subcellular distribution of GFP fluorescence was observed by confocal laser scanning microscopy (Leica) with an argon laser excitation wavelength of 488 nm and 560 nm. FM4-64 staining was performed to indicate the plasma membrane. A PUN1302 construct containing GFP was included as a control and plasmolysis was conducted by applying 1 M mannitol for 10 min.

### Rice protoplast transfection and Western blotting

Fusion proteins sp-GFP, ΔPLD-GFP and spPLD-GFP were transiently expressed in rice coleoptile protoplasts and GFP alone was expressed as a control. After transfection, the protoplasts were incubated in medium for 48 h followed by Western Blotting analysis. The incubation medium (supernatant) and protoplast homogenate (precipitation) were separated by centrifuging (2,000 g, 5 mins) and the protein of supernatant and precipitation were extracted for Western Blotting analysis using anti-GFP antibody (1:3000; Abmart).

### Protein expression and purification

Coding sequences of *Hd3a*/*Hd3a*^*M*^ (primers PET51b-*Hd3a*/*Hd3a*^*M*^-LP/RP), *RFT1*/*RFT1*^*M*^ (primers PET51b-*RFT*/*RFT1*^*M*^-LP/RP), *spPLD* (primers PET51b-*spPLD*-LP/RP) and *ΔPLD* (primers PET51b-*ΔPLD*-LP/RP) were amplified by PCR and subcloned into the *Kpn*I / *Sal*I sites of PET51b expression vector, respectively. The DNA fragments with mutated PC binding sites (*Hd3a*^*M*^ and *RFT1*^*M*^) were synthesized by Qingke company. Resultant constructs were confirmed by sequencing and transformed into *E*. *coli* strain BL21. Protein expression was induced by adding 0.6 mM IPTG (isopropyl β-D-1-thiogalactopyranoside) at 37°C for 5 h. Expressed protein was purified with Ni-NTA resin according to manufacturer’s instructions (Qiagen).

### Fat-western blotting and liposome protein association assay

Lipid-protein binding was examined by Fat-western blotting. The nitrocellulose membranes containing different lipids (PIP Strips membranes; P23751) were purchased from Thermo Fisher (Waltham, MA, USA). Nitrocellulose membranes were incubated in 3% fatty-acid-free BSA (w/v) in TBST (Tris-buffered saline, Tween 20) solution at room temperature for 1 h. After washing with TBST three times, membranes were incubated at 4°C overnight with TBST containing purified Hd3a/Hd3a^M^ or RFT1/RFT1^M^ protein. After washing three times with TBST, membranes were incubated at room temperature for 4 h with anti-His tag (Abcam) in TBST, followed by three washes with TBST and then incubated at room temperature for 1 h with secondary antibody. HRP activity was detected using LumiBest ECL reagent solution kit (Share-Bio).

Liposomal binding was performed as described [55,56] with few modifications. Briefly, Dioleoyl PC and dioleoyl PA (Avanti Polar Lipids, USA) were dissolved in chloroform and dried under nitrogen. Lipids were rehydrated in extrusion buffer (250 mM raffinose, 25 mM Tris-HCl pH 7.5, 1 mM DTT) and then extruded repeatedly by a liposome extruder to produce liposomes according to manufacturer’s instructions (Avanti Polar Lipids). Liposomes were diluted with binding buffer (25 mM Tris-HCl pH 7.5, 1.25 mM potassium chloride, 0.5 mM EDTA, 1 mM DTT), harvested by centrifugation and resuspended. Different concentrations of liposomes were incubated with purified Hd3a/Hd3a^M^ or RFT1/RFT1^M^ protein at 25°C for 45 min and centrifuged (14,000 g) for 30 min. The pellets were washed twice with binding buffer and supernatant was precipitated by addition of 1:10 (v/v) of 100% trichloroacetic acid on ice for 30 min and then centrifuged (14,000 g) for 10 min. Liposome-bound proteins in pellets and supernatant were detected by Western Blotting using anti-His antibody (1:5000; Abmart). Band density is measured by Image J.

### Assay of spPLD and ΔPLD activity

PLD activity was assayed with Phospholipase D Activity Colorimetric Assay Kit (Biovision, #K725-100). Briefly, purified spPLD or ΔPLD protein was added to assay mixture (50 μL, composed of 45 μL PLD assay buffer, 2 μL PLD enzyme mix, 2 μL PLD probe and 1 μL PLD substrate) and incubated at 25°C for 30 min, then measured absorbance at OD_570_. Activity of spPLD or ΔPLD was calculated with equation Activity = B/(ΔT×V)×D = nmol/min/mL = mU/mL. B is choline amount from Standard Curve (nmol); Δ is reaction time (min); V is sample volume added into the reaction well (mL); D is sample dilution factor.

### Lipid extraction

Total lipids were extracted according to a previous description [10] with few modifications. Ten SAM (~7 days before bolting in paddy field of Lingshui under natural SD condition) were quickly immersed in 3 mL 75°C isopropanol with 0.01% BHT (butylated hydroxytoluene) for 15 min and added with 1.5 mL chloroform and 0.6 mL water, then vortex. After shaking for 1 h at room temperature, the solvent was transferred to a new glass tube, and total 4 mL chloroform: methanol (2:1) was added and shaken for 30 min. After extraction by adding chloroform:methanol (2:1) four times, the solvent extracts were washed once with 1 mL 1 M KCl and once with 2 mL water. The solvent was evaporated under nitrogen and the lipid extract was dissolved in 2 mL chloroform. The resultant solvent extracts were used for MS analysis. The tissues after lipid extraction were dried in an oven at 105°C and dry weights were determined (3–20 mg).

### Mass spectrometric lipid analysis

Mass spectrometric lipid analysis was conducted according to previous studies [33] with few modifications. Briefly, all lipid samples extracted from SAM (with similar dry weight, 3 mg) were prepared for analysis by diluting in the solvent composition. Internal standards including 17:0–14:1-PA, 17:0–20:4-PC, 17:0–20:4-PE, 21:0–22:6-PG, 17:0–20:4-PI and 17:0–14:1-PS (from Avanti Polar Lipids) were added in each sample [57]. Samples were analyzed by flow injection using a Shimadzu CBM-20A Lite HPLC system (Kyoto, Japan) with a solvent mixture of 10 mM ammonium acetate in methanol. Mass spectrometric analysis was performed on a 5500 QTrap triple quadrupole mass spectrometer (AB Sciex, Framingham, MA, USA) equipped with a Turbo V electrospray ion source. Mass spectra were processed by the LipidView Software Version 1.1 (AB Sciex) for identification and quantification of lipids according to previous description [58]. The lipid amounts (relative ratio of total measured phospholipids) were corrected by internal standards. MultiView software (AB Sciex) built on the principle of PCA was applied to determine the statistical significance and to classify the differential groups according to previous description [33].

## Supporting information

S1 Fig Analysis at http://www.cbs.dtu.dk/services/SignalP/ reveals the high probability of a signal peptide at N-terminal (amino acid 1–23) of spPLD. (TIF)

S2 Fig Molecular phylogenetic analysis and sequence alignment revealed that signal peptide domain of spPLD is in a relatively separate evolutionary branch. A. Sequence alignment of spPLD signal peptide domain was performed with BLAST and the top 26 proteins with protein functional annotation and > 70% identity were analyzed. The bootstrap consensus tree inferred from 1000 replicates is generated to represent the evolutionary history of the taxa analyzed. The percentage of replicate trees in which the associated taxa clustered together in the bootstrap test (1000 replicates) is shown next to the branches. NCBI accession number, protein ID and species of analyzed protein are shown. B. Protein sequence of the signal peptide domain of analyzed 26 proteins in (A) was aligned with MEGA7.
(TIF)

S3 Fig Molecular phylogenetic analysis revealed that HKD motif of spPLD is highly conserved. Sequence alignment of spPLD HKD motif was performed with BLAST and the top 38 proteins with protein functional annotation and > 70% identity were analyzed. The bootstrap consensus tree inferred from 1000 replicates is generated to represent the evolutionary history of the taxa analyzed. The percentage of replicate trees in which the associated taxa clustered together in the bootstrap test (1000 replicates) is shown next to the branches. NCBI accession number, protein ID and species of analyzed protein are shown.
(TIF)

S4 Fig Sequence alignment of HKD motifs of spPLD. Protein sequence of the HKD1 motif (A) and HKD2 motif (B) of analyzed proteins (described in Figure S3) were analyzed with MEGA7.
(TIF)

S5 Fig Rhythmic expression of *spPLD* in rice leaf and secretory character of signal peptide and spPLD. A-B. Analysis using RiceXPro (https://ricexpro.dna.affrc.go.jp/) showed that *spPLD* is mainly expressed during vegetative growth before rice heading (A) and presents diurnal oscillation (B, lowest in the morning and highest in the evening). DAT, days after transplanting. C. Fluorescence observation revealed the secretory character of signal peptide and spPLD. Signal peptide of spPLD (sp), spPLD deleting signal peptide (ΔPLD) and spPLD were fused to GFP and transiently expressed in onion epidermal cells. Fluorescence of GFP or fusion proteins was observed after plasmolysis by applying 1 M mannitol for 10 min. Edges of plasma membrane were highlighted by arrows. DIC, bright field. Scale bar = 50 μm.
(TIF)

S6 Fig qPCR analysis of *spPLD* expression in *spPLDox* and *spPLD-RNAi* transgenic lines, and Δ*PLD* expression (spPLD deleting signal peptide) in Δ*PLDox* transgenic lines. Two-week-old transgenic rice seedlings were used for analysis. Expressions were normalized with ACTIN1 transcripts and relative expression levels were determined by setting expression of spPLD in ZH11 as 1.0 (there is no ΔPLD expression in ZH11). Experiments were biologically repeated three times and data are shown as mean ± SD (n = 3). Statistical analysis was performed by Tukey’s test (***, p < 0.001, compared to ZH11).
(TIF)

S7 Fig spPLD delays heading time of rice. Rice plants with altered spPLD expression were grown under natural long-day (LD, A) or short-day (SD, B) conditions and flowering plants (%) were calculated and statistically analyzed. Days to heading were scored when first panicle was bolted and fifty plants of each line were measured.
(TIF)

S8 Fig RFT1 preferentially binds to PC. A. Fat-western immunoblot analysis revealed the binding of rice RFT1 to PC, while mutation of PC binding sites (RFT1M) suppressed the binding of RFT1 to PC. Type of phospholipids of each dot is indicated. PC, phosphatidylcholine; PE, phosphatidylethanolamine; PS, phosphatidylserine; PA, phosphatidic acid; LPA, lysophosphatidic acid; LPC, lysophosphatidylcholine; S1P, sphingosine 1-phosphate; PI, phosphatidylinositol; PI3P, PI 3-monophosphate; PI4P; PI5P; PI(3,4)P2, PI 3,4-bisphosphate; PI(3,5)P2; PI(4,5)P2; PI(3,4,5)P3, phosphatidylinositol 3,4,5-trisphosphate. B. Liposome binding assay (left) and quantitative analysis (right) confirmed the preferential binding of RFT1 to PC. Purified RFT1 and RFT1M with His-tag was incubated with liposomes containing different PC:PA / PE:PA ratios. After collecting the liposomes, the portion of proteins bound to liposomes was detected by western blotting using antiHis antibody. Non-binding protein was detected in the supernatant (bottom). Band density is measured by Image J and relative density was calculated by setting the intensity under PC:PA ratio 1:1 as 1.0. Data were presented as means ± SD (n = 3).
(TIF)

S9 Fig Total contents of various phospholipids in ZH11 and rice lines with altered *spPLD* expressions, measured by mass spectrometric analysis. Examined phospholipids include: phosphatidylcholine (PC, A), phosphatidic acid (PA, B), phosphatidylethanolamine (PE, C), phosphatidylglycerol (PG, D), phosphatidylinositol (PI, E), phosphatidylserine (PS, F), lysophosphatidic acid (LPA, G), and lysophosphatidylcholine (LPC, H). Ten rice shoot apical meristem before bolting were collected and used for extracting lipids. Phospholipids were profiled by a lipidomic approach using mass spectrometry. Experiments were biologically repeated three times and data were shown as mean ± SD (n = 3). Statistical analysis was performed by Tukey’s test (*, p < 0.01; ***, p < 0.001, compared to ZH11).
(TIF)

S10 Fig Absolute amount of various PC molecular species with different saturation status in ZH11 and transgenic lines with altered *spPLD* expressions. Examined PC molecular species included: PC (32:1, A), PC (36:3, B), PC (36:2, C), (38:3, D), PC (36:5, E), PC (36:6, F), and PC (38:6, G). Ten rice shoot apical meristem before bolting were collected and used for lipids extraction. Phospholipids were profiled by a lipidomic approach using mass spectrometry. Experiments were biologically repeated three times and data were shown as mean ± SD (n = 3). Statistical analysis was performed by Tukey’s test (*, p < 0.01; ***, p < 0.001, compared to ZH11).
(TIF)

S11 Fig Tiller numbers and primary root length of ZH11 and transgenic lines with altered *spPLD* expressions. Tiller numbers (A) after fully heading and primary root length (B) of 10-day-old seedlings were calculated. Experiments were biologically repeated three times and data were shown as mean ± SD (n>20). Statistical analysis was performed by Tukey’s test (*, p < 0.01 ***, p < 0.001, compared to ZH11).
(TIF)

S12 Fig qPCR analysis of *spPLD*, *Hd3a* and *Hd3a*^*M*^ expression in transgenic *Arabidopsis*. Leaves of two-week-old transgenic Arabidopsis seedlings were used for analysis. Expressions were normalized with ACTIN2 transcripts. Experiments were biologically repeated three times and data are shown as mean ± SD (n = 3). Statistical analysis was performed by Tukey’s test (***, p < 0.001, compared to Col-0).
(TIF)

S13 Fig Expression of *spPLD* was upregulated by JA. Ten-day-old rice seedlings were treated with JA (50 or 100 μM) for different times and spPLD expression in shoot or root were examined through RT-qPCR analysis. Expression level was normalized to ACTIN1 transcript and relative expressions were calculated by setting spPLD expression under treatment at “0” as 1.0. Experiments were repeated three times and data were shown as mean ± SD (n > 3).
(TIF)

S1 Table Sequences of used primers in this study. (DOCX)
