# Supplementary figures and images for: A secretory phospholipase D hydrolyzes phosphatidylcholine to suppress rice heading time
Source: PLoS Genet. 2021 Dec 8;17(12):e1009905. doi: 10.1371/journal.pgen.1009905 (PMC8654219; doi:10.1371/journal.pgen.1009905)

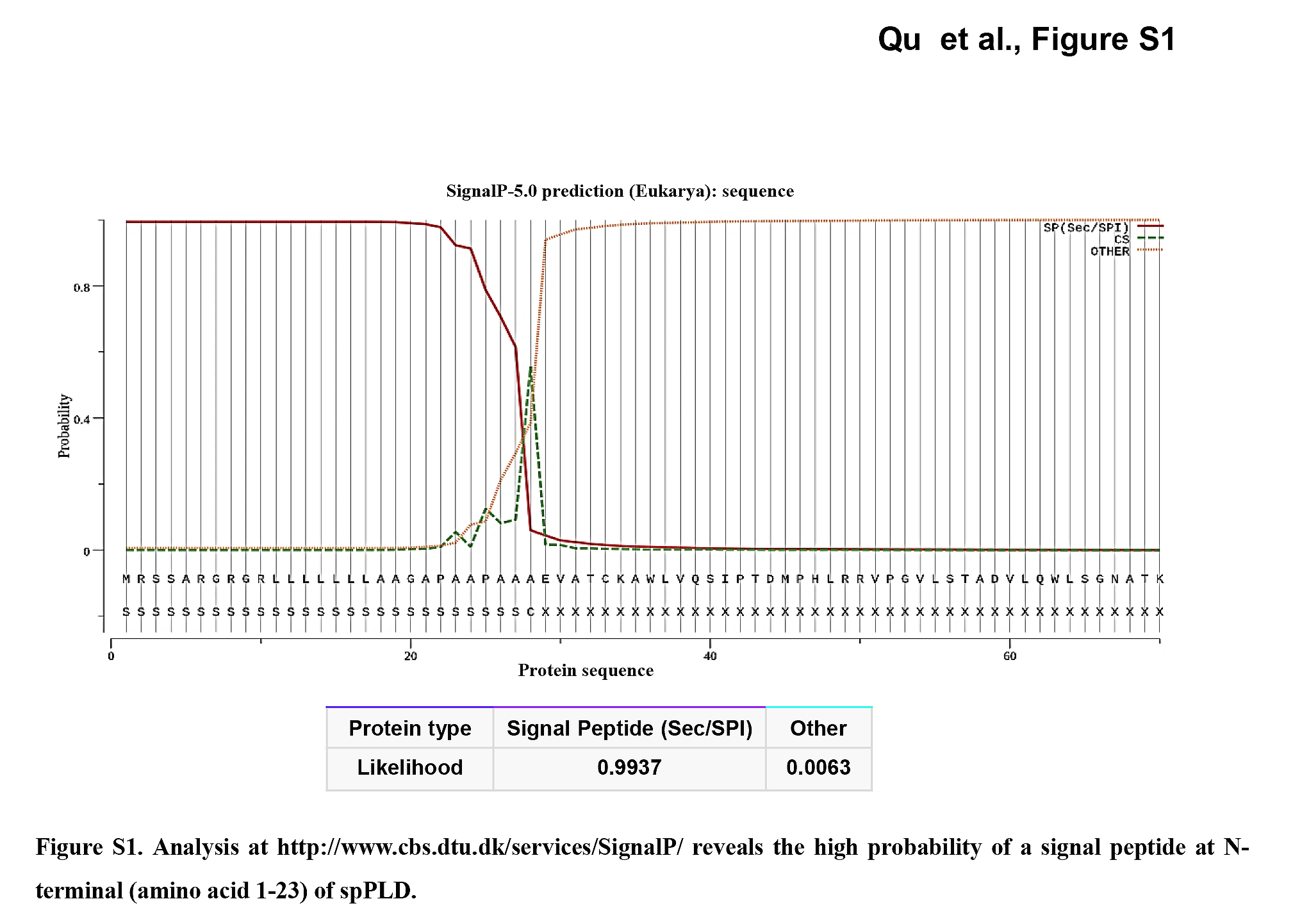

Supplement: S1 Fig — (TIF) [file pgen.1009905.s001.tif]

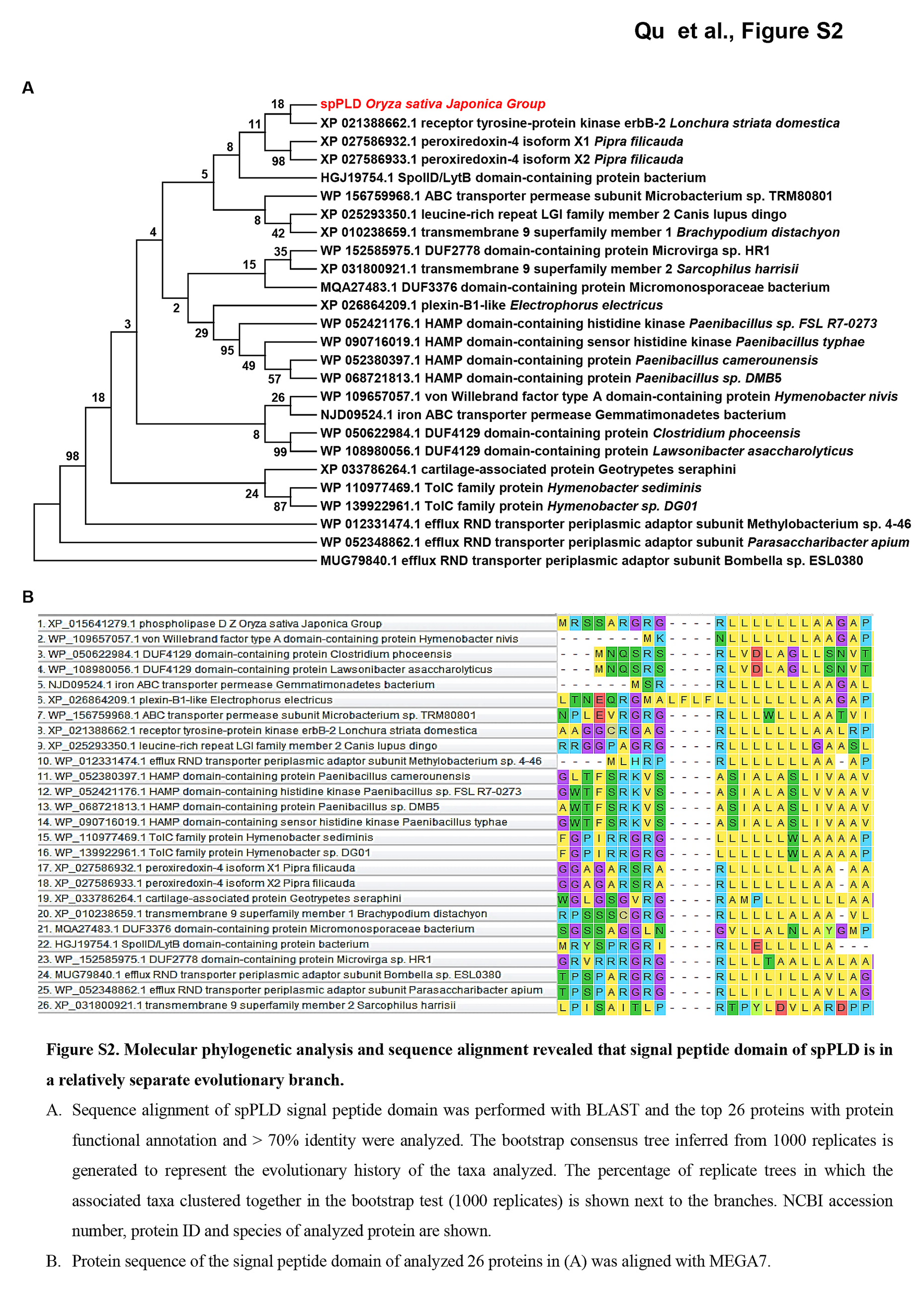

Supplement: S2 Fig — A. Sequence alignment of spPLD signal peptide domain was performed with BLAST and the top 26 proteins with protein functional annotation and > 70% identity were analyzed. The bootstrap consensus tree inferred from 1000 replicates is generated to represent the evolutionary history of the taxa analyzed. The percentage of replicate trees in which the associated taxa clustered together in the bootstrap test (1000 replicates) is shown next to the branches. NCBI accession number, protein ID and species of analyzed protein are shown. B. Protein sequence of the signal peptide domain of analyzed 26 proteins in (A) was aligned with MEGA7. (TIF) [file pgen.1009905.s002.tif]

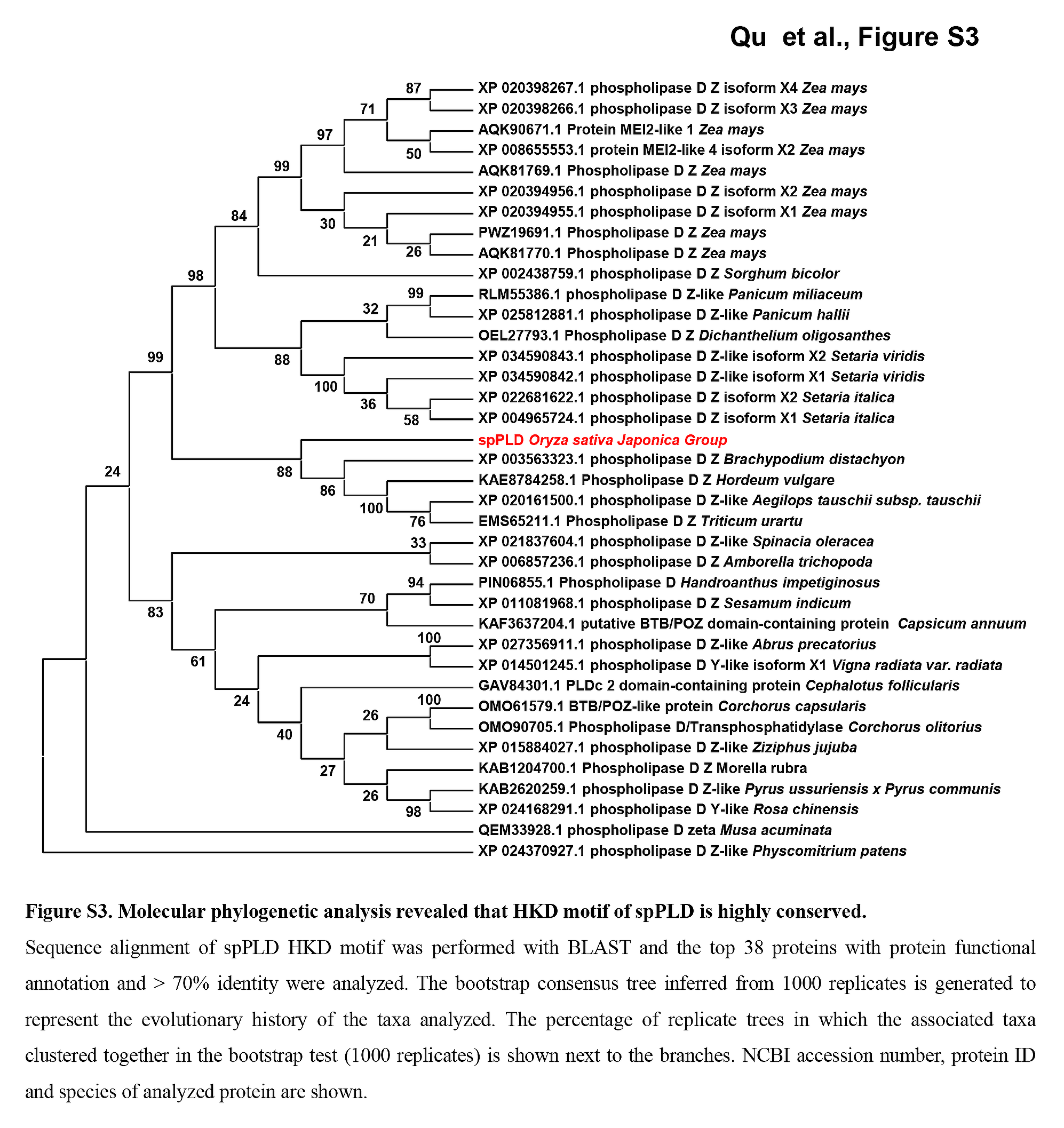

Supplement: S3 Fig — Sequence alignment of spPLD HKD motif was performed with BLAST and the top 38 proteins with protein functional annotation and > 70% identity were analyzed. The bootstrap consensus tree inferred from 1000 replicates is generated to represent the evolutionary history of the taxa analyzed. The percentage of replicate trees in which the associated taxa clustered together in the bootstrap test (1000 replicates) is shown next to the branches. NCBI accession number, protein ID and species of analyzed protein are shown. (TIF) [file pgen.1009905.s003.tif]

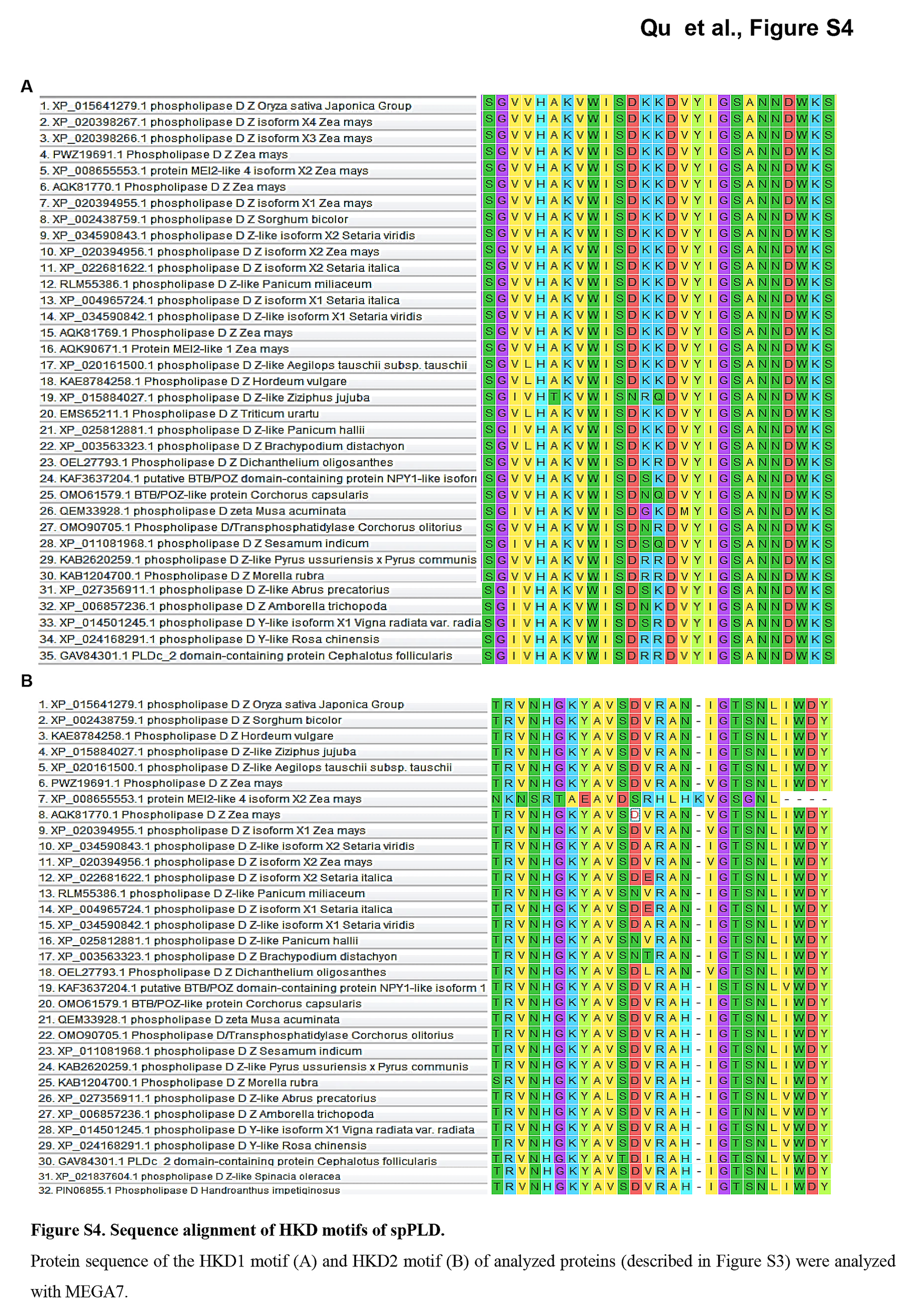

Supplement: S4 Fig — Protein sequence of the HKD1 motif (A) and HKD2 motif (B) of analyzed proteins (described in Figure S3) were analyzed with MEGA7. (TIF) [file pgen.1009905.s004.tif]

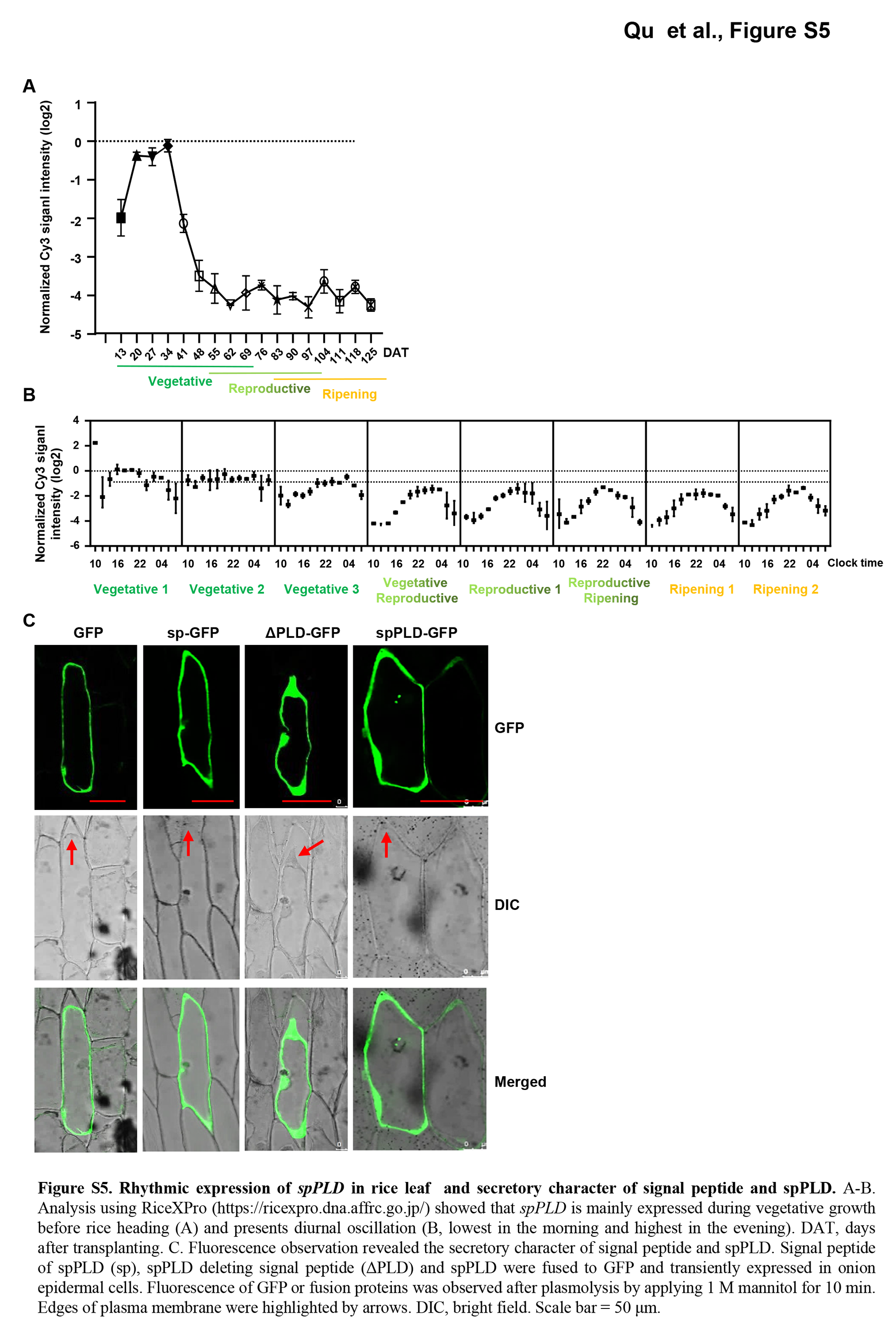

Supplement: S5 Fig — A-B. Analysis using RiceXPro (https://ricexpro.dna.affrc.go.jp/) showed that spPLD is mainly expressed during vegetative growth before rice heading (A) and presents diurnal oscillation (B, lowest in the morning and highest in the evening). DAT, days after transplanting. C. Fluorescence observation revealed the secretory character of signal peptide and spPLD. Signal peptide of spPLD (sp), spPLD deleting signal peptide (ΔPLD) and spPLD were fused to GFP and transiently expressed in onion epidermal cells. Fluorescence of GFP or fusion proteins was observed after plasmolysis by applying 1 M mannitol for 10 min. Edges of plasma membrane were highlighted by arrows. DIC, bright field. Scale bar = 50 μm. (TIF) [file pgen.1009905.s005.tif]

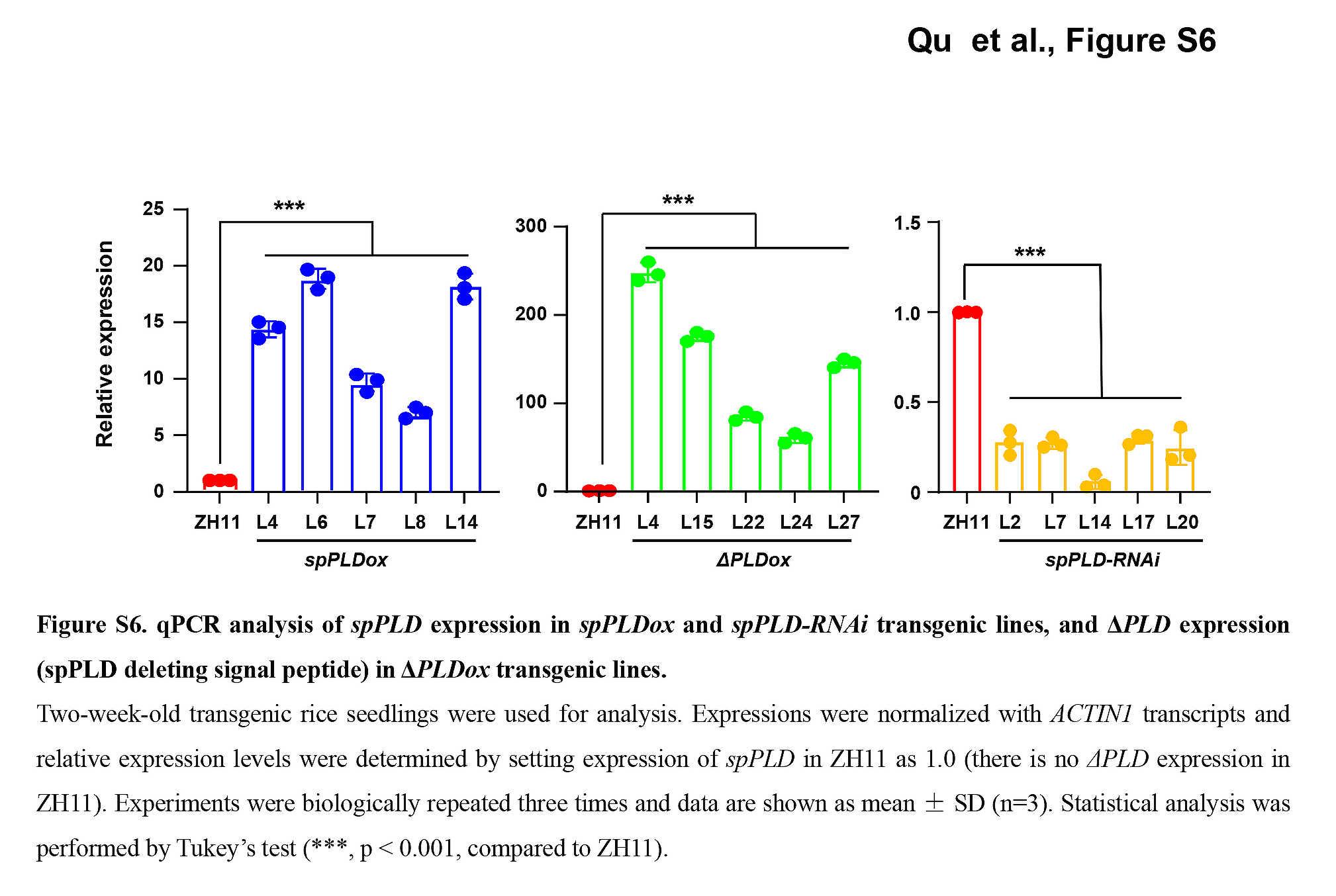

Supplement: S6 Fig — Two-week-old transgenic rice seedlings were used for analysis. Expressions were normalized with ACTIN1 transcripts and relative expression levels were determined by setting expression of spPLD in ZH11 as 1.0 (there is no ΔPLD expression in ZH11). Experiments were biologically repeated three times and data are shown as mean ± SD (n = 3). Statistical analysis was performed by Tukey’s test (***, p < 0.001, compared to ZH11). (TIF) [file pgen.1009905.s006.tif]

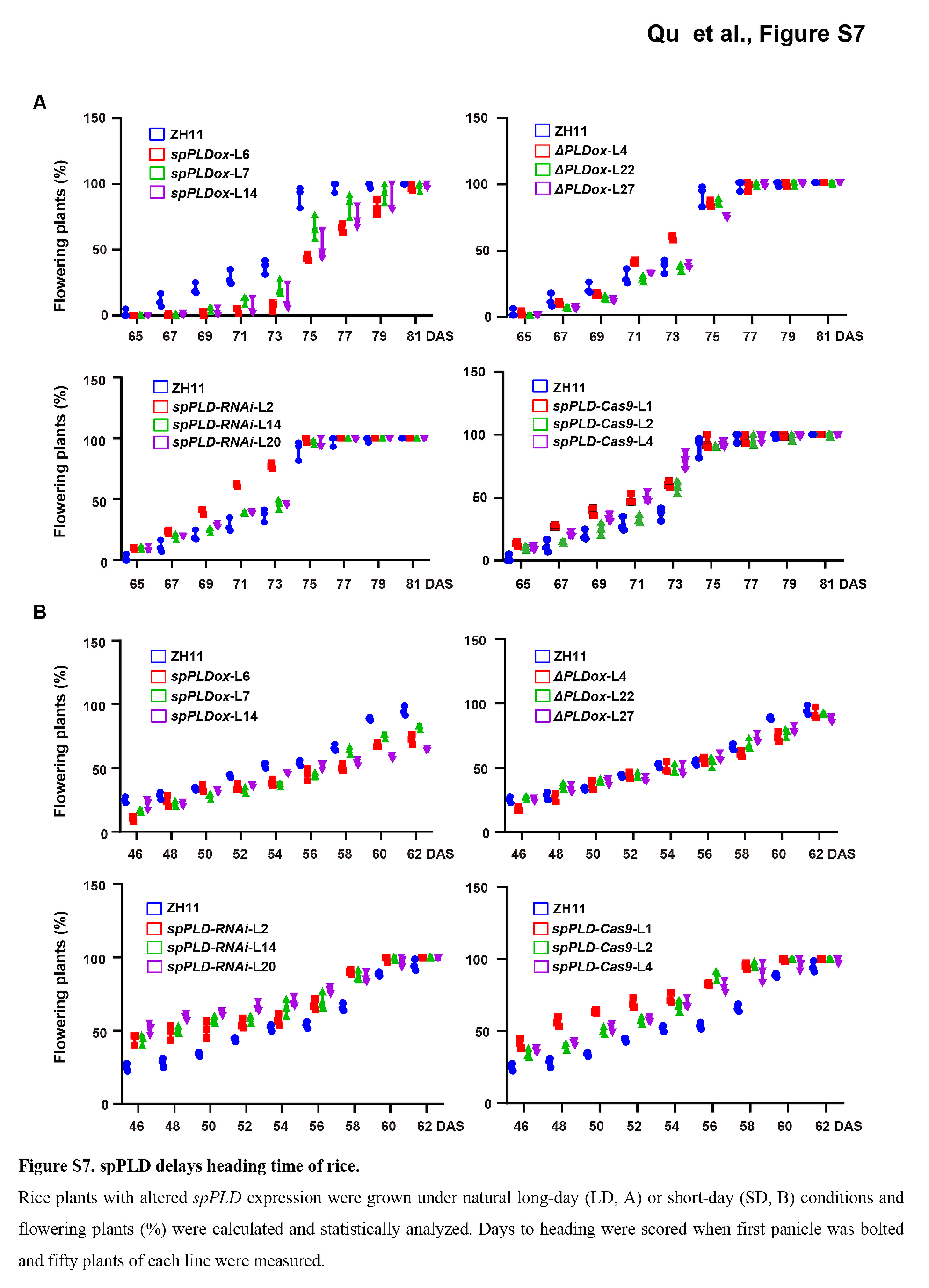

Supplement: S7 Fig — Rice plants with altered spPLD expression were grown under natural long-day (LD, A) or short-day (SD, B) conditions and flowering plants (%) were calculated and statistically analyzed. Days to heading were scored when first panicle was bolted and fifty plants of each line were measured. (TIF) [file pgen.1009905.s007.tif]

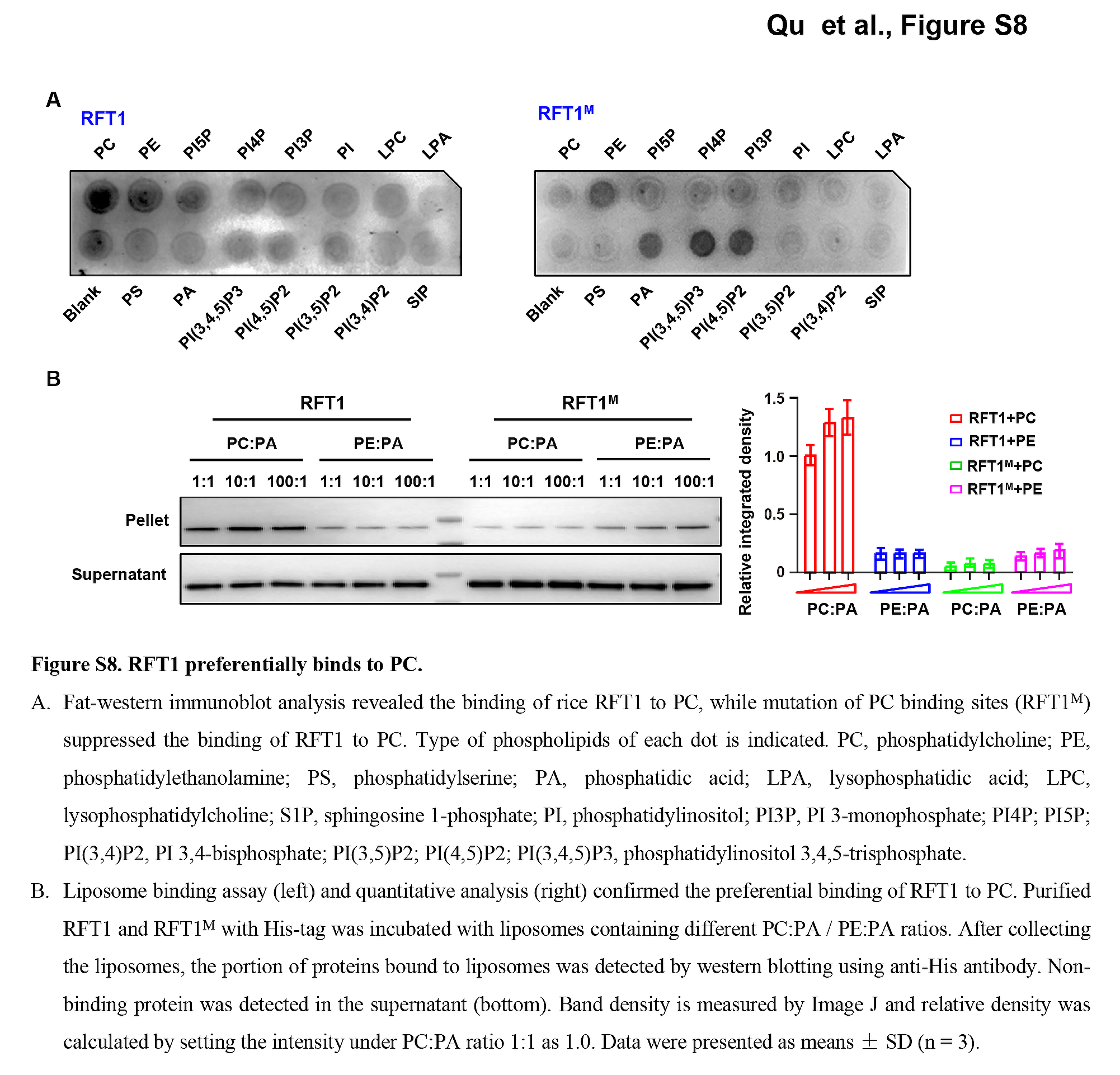

Supplement: S8 Fig — A. Fat-western immunoblot analysis revealed the binding of rice RFT1 to PC, while mutation of PC binding sites (RFT1M) suppressed the binding of RFT1 to PC. Type of phospholipids of each dot is indicated. PC, phosphatidylcholine; PE, phosphatidylethanolamine; PS, phosphatidylserine; PA, phosphatidic acid; LPA, lysophosphatidic acid; LPC, lysophosphatidylcholine; S1P, sphingosine 1-phosphate; PI, phosphatidylinositol; PI3P, PI 3-monophosphate; PI4P; PI5P; PI(3,4)P2, PI 3,4-bisphosphate; PI(3,5)P2; PI(4,5)P2; PI(3,4,5)P3, phosphatidylinositol 3,4,5-trisphosphate. B. Liposome binding assay (left) and quantitative analysis (right) confirmed the preferential binding of RFT1 to PC. Purified RFT1 and RFT1M with His-tag was incubated with liposomes containing different PC:PA / PE:PA ratios. After collecting the liposomes, the portion of proteins bound to liposomes was detected by western blotting using antiHis antibody. Non-binding protein was detected in the supernatant (bottom). Band density is measured by Image J and relative density was calculated by setting the intensity under PC:PA ratio 1:1 as 1.0. Data were presented as means ± SD (n = 3). (TIF) [file pgen.1009905.s008.tif]

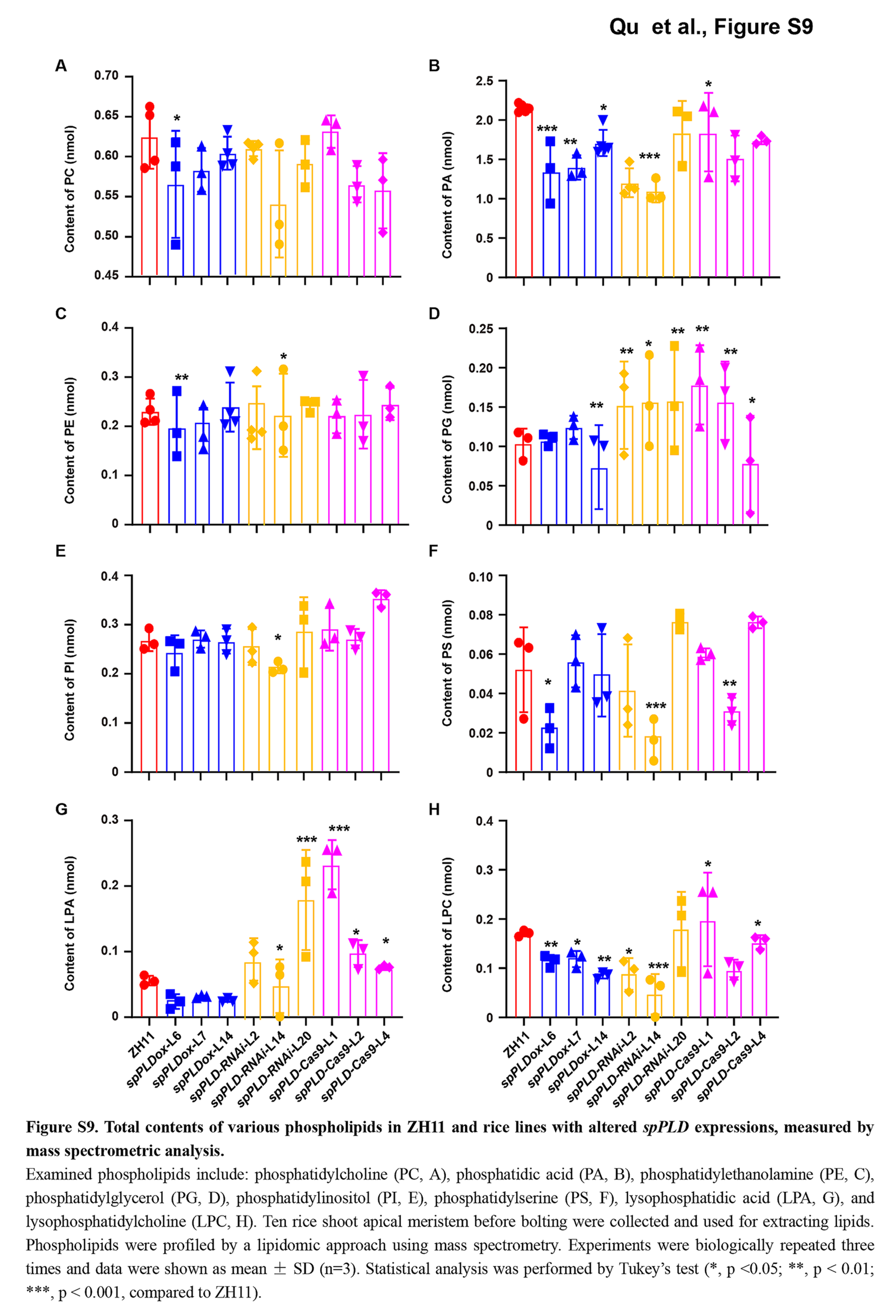

Supplement: S9 Fig — Examined phospholipids include: phosphatidylcholine (PC, A), phosphatidic acid (PA, B), phosphatidylethanolamine (PE, C), phosphatidylglycerol (PG, D), phosphatidylinositol (PI, E), phosphatidylserine (PS, F), lysophosphatidic acid (LPA, G), and lysophosphatidylcholine (LPC, H). Ten rice shoot apical meristem before bolting were collected and used for extracting lipids. Phospholipids were profiled by a lipidomic approach using mass spectrometry. Experiments were biologically repeated three times and data were shown as mean ± SD (n = 3). Statistical analysis was performed by Tukey’s test (*, p < 0.01; ***, p < 0.001, compared to ZH11). (TIF) [file pgen.1009905.s009.tif]

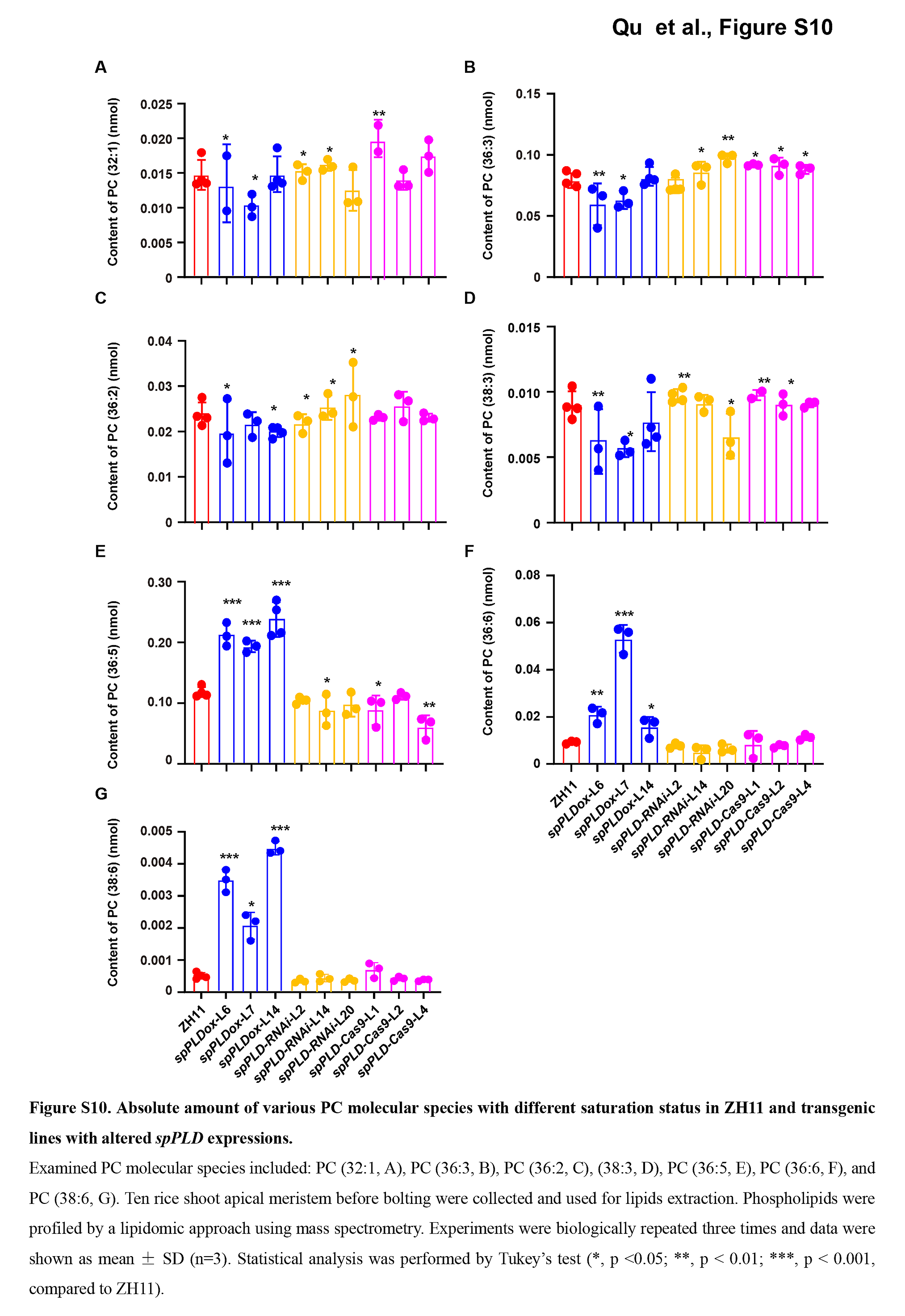

Supplement: S10 Fig — Examined PC molecular species included: PC (32:1, A), PC (36:3, B), PC (36:2, C), (38:3, D), PC (36:5, E), PC (36:6, F), and PC (38:6, G). Ten rice shoot apical meristem before bolting were collected and used for lipids extraction. Phospholipids were profiled by a lipidomic approach using mass spectrometry. Experiments were biologically repeated three times and data were shown as mean ± SD (n = 3). Statistical analysis was performed by Tukey’s test (*, p < 0.01; ***, p < 0.001, compared to ZH11). (TIF) [file pgen.1009905.s010.tif]

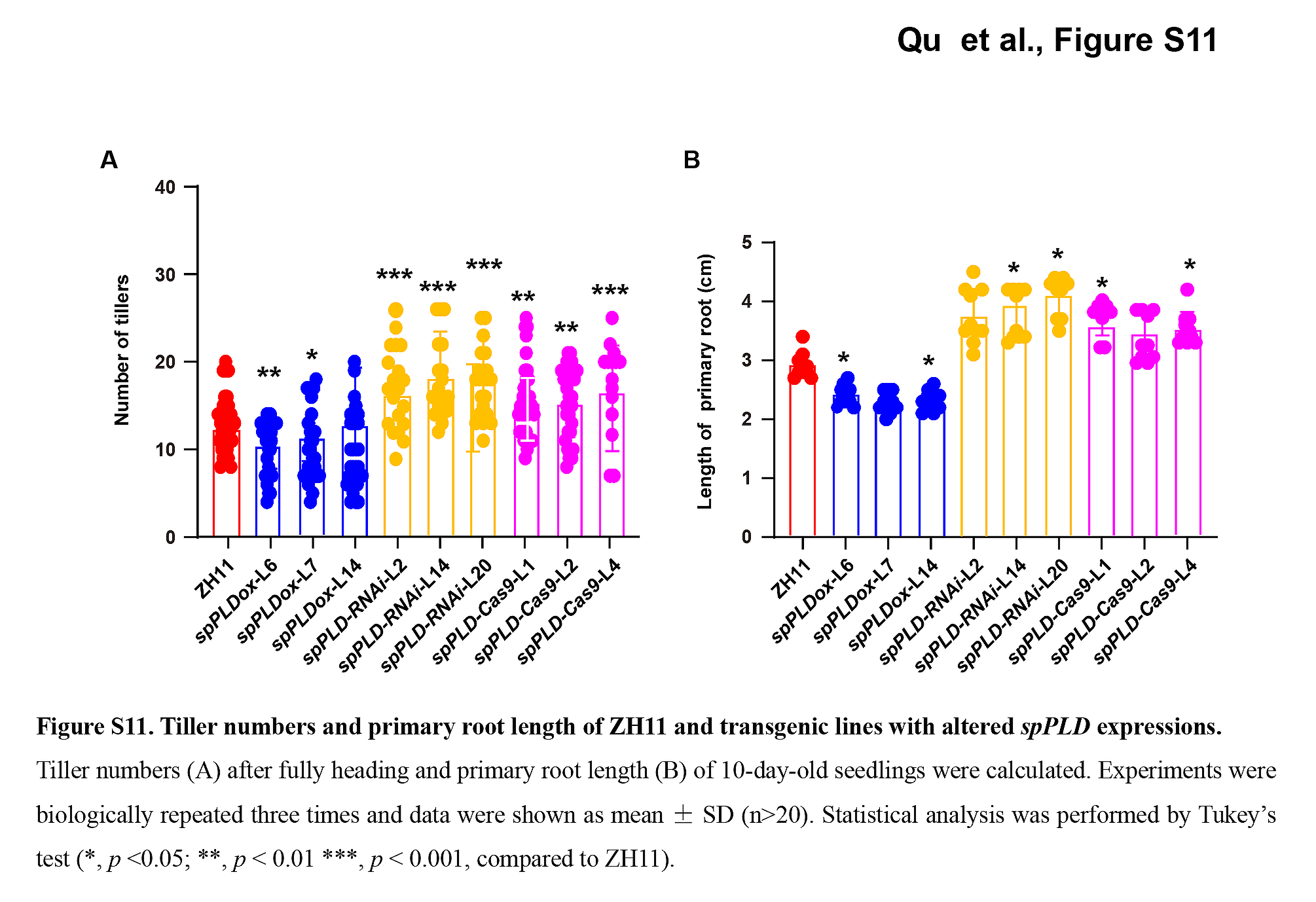

Supplement: S11 Fig — Tiller numbers (A) after fully heading and primary root length (B) of 10-day-old seedlings were calculated. Experiments were biologically repeated three times and data were shown as mean ± SD (n>20). Statistical analysis was performed by Tukey’s test (*, p < 0.01 ***, p < 0.001, compared to ZH11). (TIF) [file pgen.1009905.s011.tif]

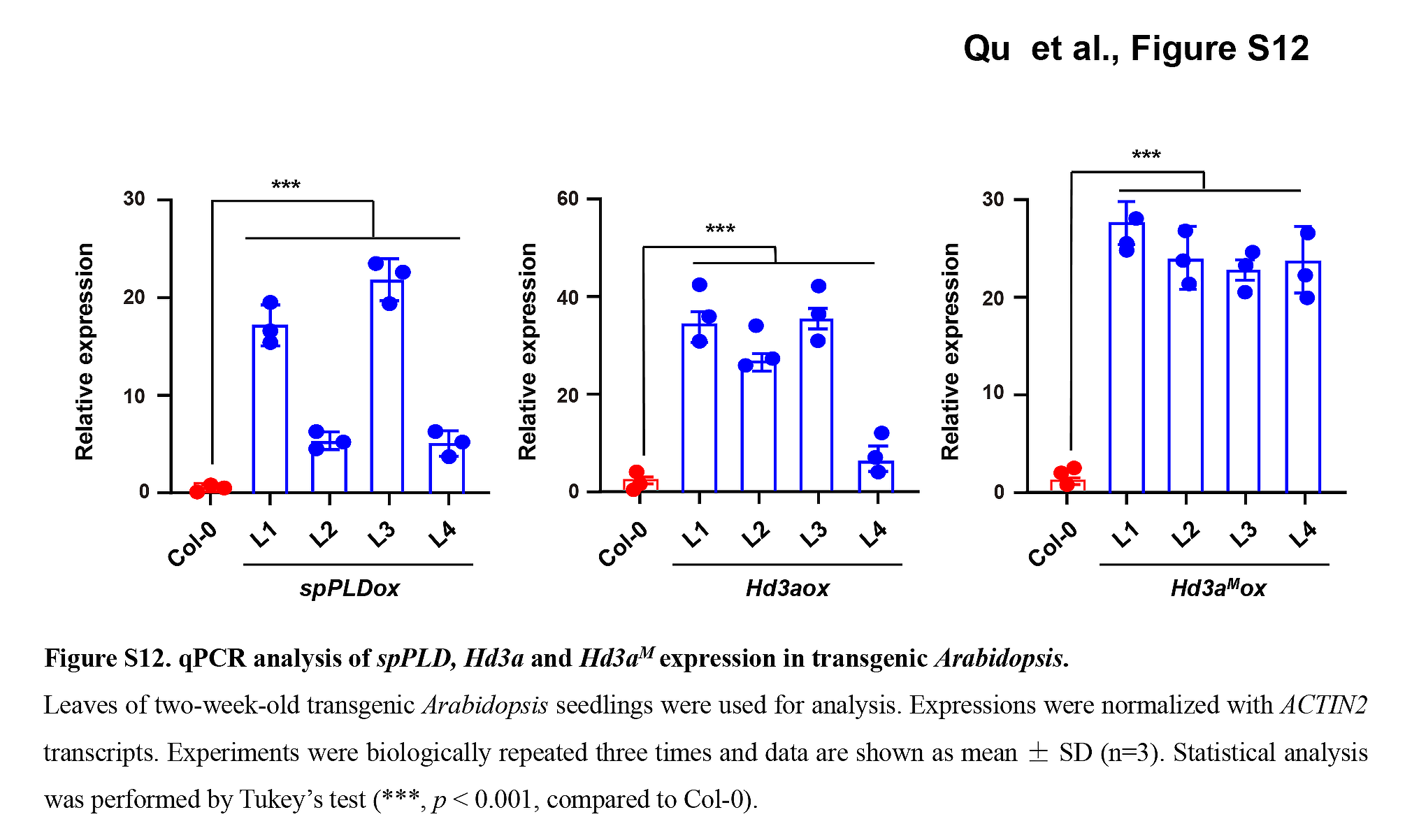

Supplement: S12 Fig — Leaves of two-week-old transgenic Arabidopsis seedlings were used for analysis. Expressions were normalized with ACTIN2 transcripts. Experiments were biologically repeated three times and data are shown as mean ± SD (n = 3). Statistical analysis was performed by Tukey’s test (***, p < 0.001, compared to Col-0). (TIF) [file pgen.1009905.s012.tif]

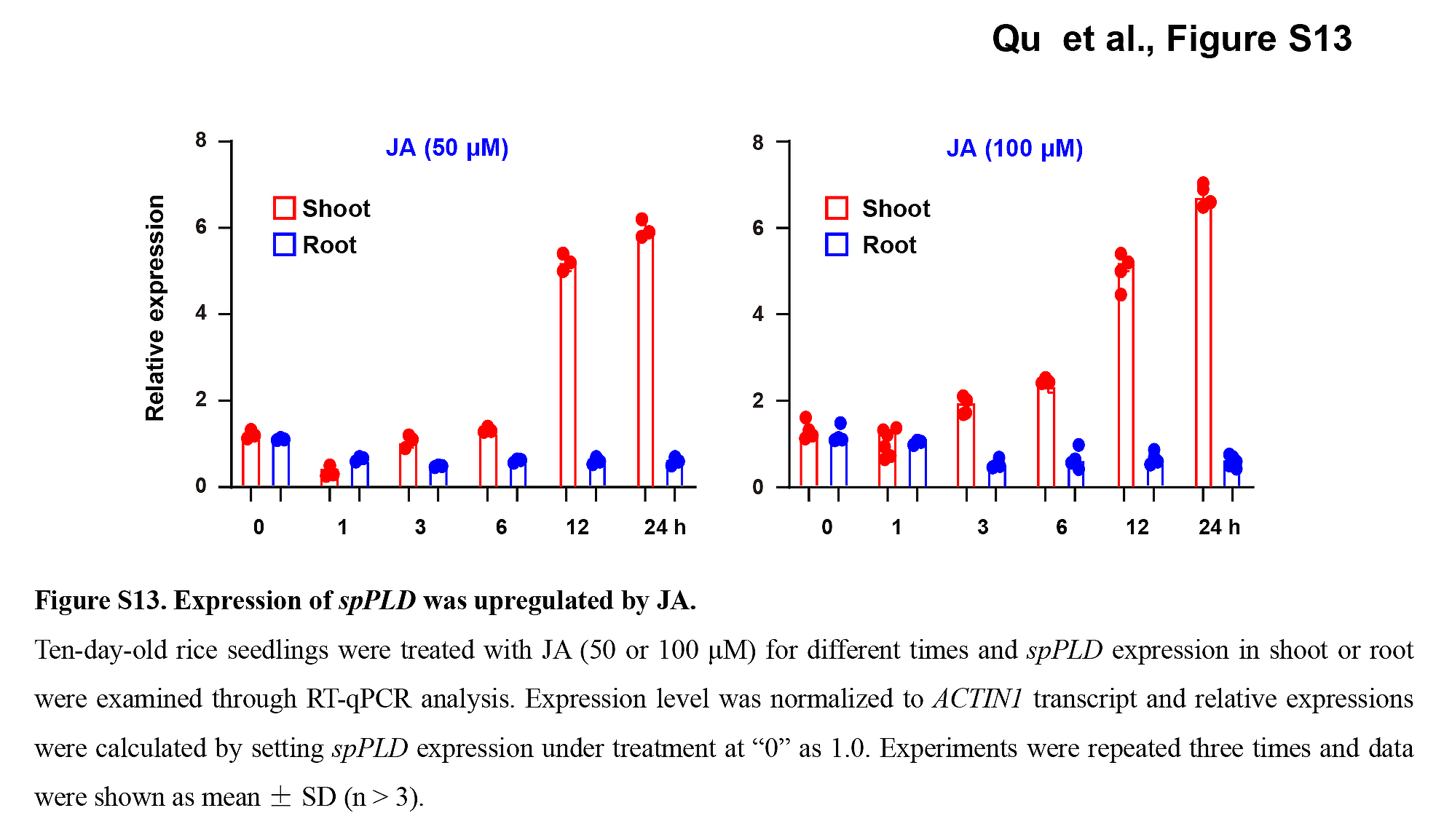

Supplement: S13 Fig — Ten-day-old rice seedlings were treated with JA (50 or 100 μM) for different times and spPLD expression in shoot or root were examined through RT-qPCR analysis. Expression level was normalized to ACTIN1 transcript and relative expressions were calculated by setting spPLD expression under treatment at “0” as 1.0. Experiments were repeated three times and data were shown as mean ± SD (n > 3). (TIF) [file pgen.1009905.s013.tif]
