# Supplementary material for: A secretory phospholipase D hydrolyzes phosphatidylcholine to suppress rice heading time
Source: PLoS Genet. 2021 Dec 8;17(12):e1009905. doi: 10.1371/journal.pgen.1009905 (PMC8654219; doi:10.1371/journal.pgen.1009905)
Supplement: S1 Table — (DOCX) [file pgen.1009905.s014.docx]

**S1 Table. Sequences of used primers in this study.** Added restriction enzymes are underlined.

| **Name** | **Sequence (5'-3')** | **Restriction enzymes** |
| --- | --- | --- |
| PUN1301-*spPLD*-LP | CGGGGTACCATGCGTTCCTCCGCCCGCGGC | *Kpn*I |
| PUN1301-*spPLD*-RP | CGGGGTACCTTATGCAGATGCTTCCAGTGG | *Kpn*I |
| PUN1301-*ΔPLD*-LP | CGGGGTACCATGGCGCCGGCGGCGGCGGAG | *Kpn*I |
| PUN1301-*ΔPLD*-RP | CGGGGTACCTTATGCAGATGCTTCCAGTGG | *Kpn*I |
| At-*ΔPLD*-PHB-LP | CCGCTCGAGATGGCGCCGGCGGCGGCGGAG | *Xho*I |
| At-*ΔPLD*-PHB-RP | TGCTCTAGATTATGCAGATGCTTCCAGTGG | *Xba*I |
| At-*spPLD*-PHB-LP | CCGCTCGAGATGCGTTCCTCCGCCCGCGGC | *Xho*I |
| At-*spPLD*-PHB-RP | TGCTCTAGATTATGCAGATGCTTCCAGTGG | *Xba*I |
| At- *Hd3a* -PHB-LP | CGGGGTACCATGGCCGGAAGTGGCAGGGACA | *Kpn*I |
| At- *Hd3a* -PHB-RP | TGCTCTAGACTAGGGGTAGACCCTCCTGCCG | *Xba*I |
| At- *Hd3a^M^* -PHB-LP | CGGGGTACCATGGCCGGAAGTGGCAGGGACA |  |
| At- *Hd3a^M^* -PHB-RP | TGCTCTAGACTAGGGGTAGACCCTCCTGCCG |  |
| *sp*-GFP-LP | ACGCGTCGACATGCGTTCCTCCGCCCGCGGC | *Sal*I |
| *sp*-GFP-RP | GACTAGTCCCGCCGGCGCACCGGCGGCGA | *Spe*I |
| *ΔPLD*-GFP-LP | ACGCGTCGACATGGCGCCGGCGGCGGCGGAG | *Sal*I |
| *ΔPLD*-GFP-RP | GACTAGTCCTGCAGATGCTTCCAGTGG | *Spe*I |
| *spPLD*-GFP-LP | ACGCGTCGACATGCGTTCCTCCGCCCGCGGC | *Sal*I |
| *spPLD*-GFP-RP | GACTAGTCCTGCAGATGCTTCCAGTGG | *Spe*I |
| PUN1302-*spPLD*-LP | GAAGATCTATGCGTTCCTCCGCCCGCGGC | *Bgl*II |
| PUN1302-*spPLD*-RP | GACTAGTCCTGCAGATGCTTCCAGTGG | *Spe*I |
| *spPLD-RNAi*-LP | TCCCCGCGGGGCCGCGGCCGCGGGCG | *Sac*I |
| *spPLD-RNAi*-RP | AACTGCAGGGGACGCGCCGCAGGTGG | *Pst*I |
| PET51b-*Hd3a*-LP | CGGGGTACCATGGCCGGAAGTGGCAGGGACA | *Kpn*I |
| PET51b-*Hd3a*-RP | GCGTCGACCTAGGGGTAGACCCTCCTGCCG | *Sal*I |
| PET51b-*Hd3a^M^*-LP | CGGGGTACCATGGCCGGAAGTGGCAGGGACA | *Kpn*I |
| PET51b-*Hd3a^M^*-RP | GCGTCGACCTAGGGGTAGACCCTCCTGCCG | *Sal*I |
| PET51b-*RFT*-LP | GTCGACATGATGGACTGCGATAAACAGTTGTT | *Sal*I |
| PET51b-*RFT*-RP | AGCTTTGCAAGTTTGTCGCGGCTCTT | *Hind* III |
| PET51b-*RFT^M^*-LP | GTCGACATGATGGACTGCGATAAACAGTTGTT | *Sal*I |
| PET51b-*RFT^M^*-RP | AGCTTTGCAAGTTTGTCGCGGCTCTT | *Hind* III |
| PET51b-*spPLD*-LP | CGGGGTACCATGCGTTCCTCCGCCCGCGGCC | *Kpn*I |
| PET51b-*spPLD*-RP | CGGGGTACCTGCAGATGCTTCCAGTGGT | *Sal*I |
| PET51b-*ΔPLD*-LP | CGGGGTACCGGCGGCGGCGGAGGTGGCCAC | *Kpn*I |
| PET51b-*ΔPLD*-RP | CGGGGTACCTGCAGATGCTTCCAGTGGT | *Sal*I |
| *sgRNA-Cas9* | ATGTGCTGCAATGGCTATCT |  |
| *spPLD-Cas9*-LP | GGCAATGTGCTGCAATGGCTATCT |  |
| *spPLD-Cas9*-RP | AAACAGATAGCCATTGCAGCACAT |  |
| *ACTIN1*-LP | TGAGTAACCACGCTCCGTCA |  |
| *ACTIN1*-RP | CCTTCAACACCCCTGCTATG |  |
| *ACTIN2*-LP | CCGGTATTGTGCTCGATTCTG |  |
| *ACTIN2*-RP | TTCCCGTTCTGCGGTAGTGG |  |
| RT-*spPLD*-LP | AAGGTTCGGAGCTGACAAGG |  |
| RT-*spPLD*-RP | TTGTCCCAGGCAACTTTGGT |  |
| RT-*Hd3a*-LP | GCCGGAAGTGGCAGGGACA |  |
| RT-*Hd3a*-RP | GGGTAGACCCTCCTGCCG |  |
| RT-*ΔPLD*-LP | TCAAATTCGGGGGAGTGGTG |  |
| RT-*ΔPLD*-RP | TCGGTGTTCGGGATGAAGTG |  |
| RT-*OsMADS14*-LP | CGGTTGCGAGACGAGGAA |  |
| RT-*OsMADS14*-RP | GAAAGACGGTGCTGGACGAA |  |
| RT-*OsMADS15*-LP | CGTCGTCGGCCAAACAG |  |
| RT-*OsMADS15*-RP | TGACTTCAATTCATTCAAGGTTGCT |  |
| RT-*OsMADS18*-LP | AGCCAAATACTGAGGACC |  |
| RT-*OsMADS18*-RP | TTGCTGGAGTTCTTTTATTGT |  |
| RT-*OsMADS34*-LP | CAACCAGAGCACTTCTTCCA |  |
| RT-*OsMADS34*-RP | CTGAAGCTGAAACGGTAGCT |  |
